# MicroRNA-224 down-regulates Glycine *N*-methyltransferase gene expression in Hepatocellular Carcinoma

**DOI:** 10.1038/s41598-018-30682-5

**Published:** 2018-08-16

**Authors:** Jung-Hsien Hung, Chung-Hsien Li, Ching-Hua Yeh, Pin-Cheng Huang, Cheng-Chieh Fang, Yen-Fu Chen, Kuo-Jui Lee, Chih-Hung Chou, Hsin-Yun Cheng, Hsien-Da Huang, Marcelo Chen, Ting-Fen Tsai, Anya Maan-Yuh Lin, Chia-Hung Yen, Ann-Ping Tsou, Yu-Chang Tyan, Yi-Ming Arthur Chen

**Affiliations:** 1 Department and Institute of Pharmacology, National Yang-Ming University, Taipei, Taiwan; 2 Center for Infectious Disease and Cancer Research (CICAR), Kaohsiung Medical University, Kaohsiung, Taiwan; 3 Institute of Bioinformatics and Systems Biology, National Chiao Tung University, HsinChu, Taiwan; 4 Department of Biological Science and Technology, National Chiao Tung University, HsinChu, Taiwan; 5 Department of Urology, Mackay Memorial Hospital, Taipei, Taiwan; 6 School of Medicine, Mackay Medical College, New Taipei City, Taiwan; 7 Department of Cosmetic Applications and Management, Mackay Junior College of Medicine, Nursing and Management, Taipei, Taiwan; 8 Department of Life Sciences and Institute of Genome Sciences, National Yang-Ming University, Taipei, Taiwan; 9 Department of Medical Research, Taipei Veterans General Hospital, Taipei, Taiwan; 10 Faculty of Pharmacy, Taipei, Taiwan, National Yang-Ming University, Taipei, Taiwan; 11 Graduate Institute of Natural Products, College of Pharmacy, Kaohsiung Medical University, Kaohsiung, Taiwan; 12 Graduate Institute of Medical Sciences, Taipei Medical University, Taipei, Taiwan; 13 Gaduate Institute of Medicine, Kaohsiung Medical University, Kaohsiung, Taiwan; 14 Institute of Biomedical Sciences, National Sun Yat-sen University, Kaohsiung, Taiwan

## Abstract

Glycine *N*-methyltransferase (GNMT) is a tumor suppressor for HCC. It is down-regulated in HCC, but the mechanism is not fully understood. MicroRNA-224 (miR-224) acts as an onco-miR in HCC. This study is the first to investigate miR-224 targeting the coding region of GNMT transcript. The GNMT-MT plasmid containing a miR-224 binding site silent mutation of the GNMT coding sequence can escape the suppression of miR-224 in HEK293T cells. Expression of both exogenous and endogenous GNMT was suppressed by miR-224, while miR-224 inhibitor enhanced GNMT expression. miR-224 counteracts the effects of GNMT on the reduction of cell proliferation and tumor growth. The levels of miR-224 and GNMT mRNA showed a significant inverse relationship in tumor specimens from HCC patients. Utilizing CCl4-treated hepatoma cells and mice as a cell damage of inflammatory or liver injury model, we observed that the decreased expression levels of GNMT were accompanied with the elevated expression levels of miR-224 in hepatoma cells and mouse liver. Finally, hepatic AAV-mediated GNMT also reduced CCl4-induced miR-224 expression and liver fibrosis. These results indicated that AAV-mediated GNMT has potential liver protection activity. miR-224 can target the GNMT mRNA coding sequence and plays an important role in GNMT suppression during liver tumorigenesis.

## Introduction

Hepatocellular carcinoma (HCC) was the fifth most frequently diagnosed cancer and the second most frequent cause of cancer death in men worldwide in 2012^1^. In the clinic, chronic liver failure is a risk factor of hepatic fibrosis and cirrhosis. Many factors can contribute to initiate or develop chronic liver failure for final results of liver cirrhosis or HCC. Causes of liver diseases in our life may include alcohol consumption, infection, toxic exposure, metabolic syndrome or a genetic defect^2^. Effects of these factors on the liver may include inflammation, scarring, obstructions, blood clotting abnormalities, and liver failure.

Glycine N-methyltransferase (GNMT, EC2.1.1.20) is a protein with multiple functions^3^. It not only plays an important role in the regulation of the hepatic S-adenosylmethionine pool, but also serves as a folate-binding protein. In addition, GNMT binds carcinogens such as polyaromatic hydrocarbons and aflatoxins, and prevents DNA adduct formation and cytotoxicity induced by these carcinogens. In 1998, we were the first group to report that GNMT was down-regulated in both human HCC tumor tissues and tumor cell lines^4^. Subsequently, we used genotypic analysis to show that the rates of loss of heterozygosity at the GNMT locus in paired tumor and tumor-adjacent tissues from HCC patients were 36 to 47%^5^. These suggest that GNMT gene expression is frequently repressed in HCC, but the mechanism has not been fully elucidated. Using GNMT knockout mouse models (*Gnmt*^−/−^) we and another group demonstrated that *Gnmt*^−/−^ mice of both genders develop HCC spontaneously at high rates and the pathology of the liver tumors mimics the multiple stages of human HCC development, including chronic hepatitis, fatty nodules, hemangioma, dysplastic nodules and HCC^6–8^. In addition, we demonstrated that the global DNA hypomethylation and aberrant expression of DNA methyltransferases 1 and 3b were associated with the tumorigenesis in *Gnmt*^−/−^ mice^7^. It is crucial to recognize that GNMT regulates the mTOR and ERK1/2 pathways by interacting with DEP domain containing mTOR-interacting protein (DEPTOR) and Niemann–Pick type C2 protein in HCC tumorigenesis, respectively^9,10^. Recently, we identified a novel PTEN inhibitor-PREX2 as another GNMT-interacting protein. We showed that such interaction enhanced degradation of PREX2 through an E3 ligase HectH9-mediated proteasomal ubiquitination pathway^11^. These studies implicate that GNMT is actively involved in maintaining normal liver function and further support the role of GNMT as a tumor suppressor gene in HCC.

MicroRNAs are a class of small non-coding single-stranded RNAs that regulate post-transcriptional gene expression through mRNA degradation and translational repression of mRNA in mammalian cells^12^. Most microRNAs bind to their targets at the 3′untranslated region (3′UTR). However, a recent study showed that microRNA can also target at the coding sequences (CDS)^13,14^. MicroRNAs have been predicted to regulate the expression of approximately 30% of fundamental genes^15^. Moreover, studies indicated that microRNAs act as oncogenes (onco-miRs) or tumor suppressors in tumorigenesis^16^. A number of microRNAs have been reported to be dysregulated in HCC^17^.

Among them, miR-224 as an onco-miR plays a role in HCC development and progression based on the fact that it binds to 3′UTR regions of multiple genes that are involved in apoptosis, cell proliferation, migration, invasion and autophagy^18–24^. Here, we show that GNMT is also under miR-224 (onco-miR) regulation in HCC. We found that human miR-224 targeted the coding region of GNMT mRNA and enhanced cell proliferation. Therefore, we hypothesized that GNMT is regulated by miR-224 in tumorigenesis of HCC.

## Results

### Identification of miR-224 as a putative target of GNMT

**Figure 1:**
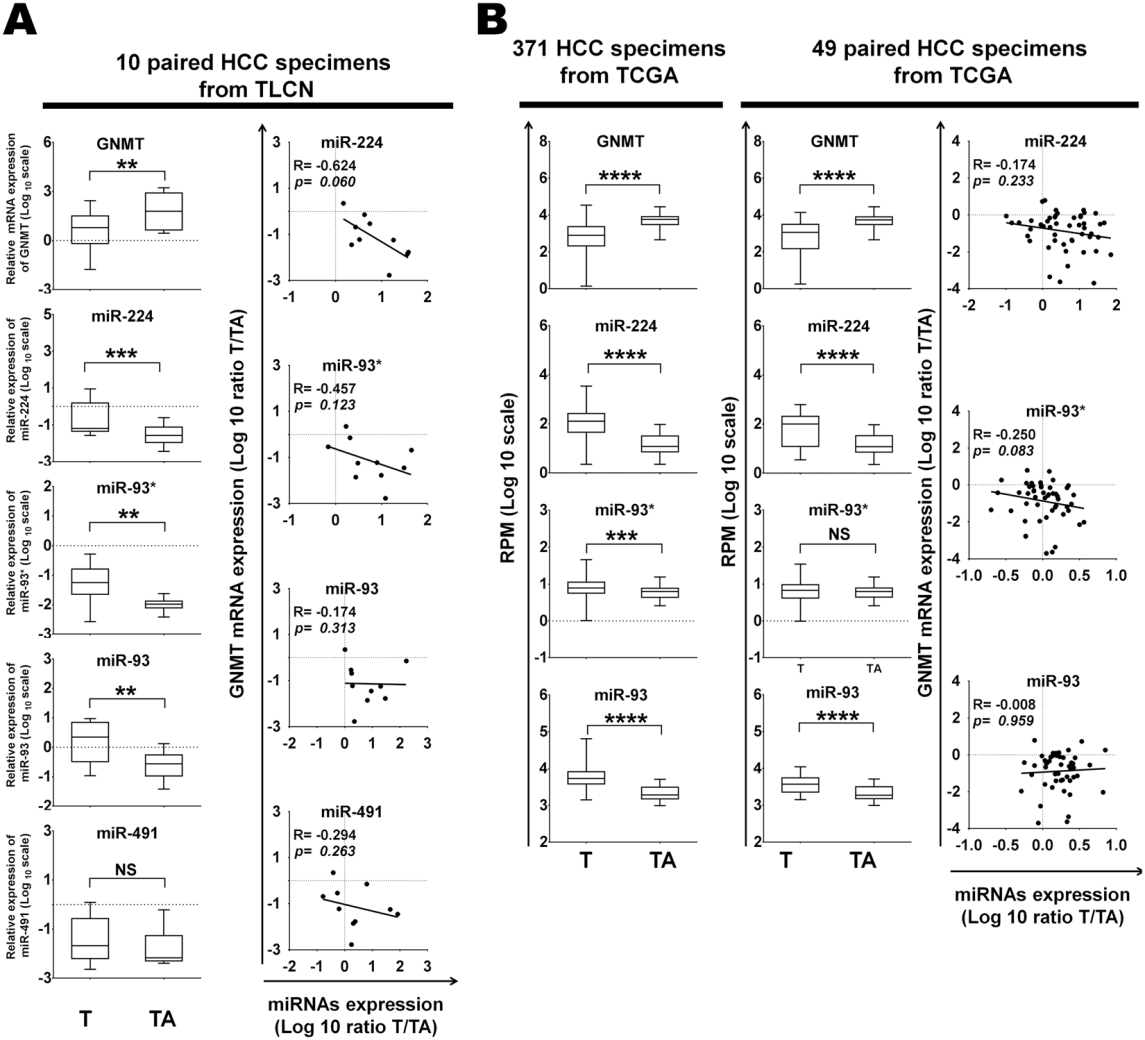
Identification of miR-224 as a putative target of GNMT. (**A**) The GNMT and four miRs (miR-224, -93*, -93, and -491) expression levels were quantified by RT-qPCR. The relative expression fold of GNMT and miR-224 were normalized to TBP (TATA-binding protein) and RNU48 (left panel). The horizontal lines inside the box represent the medians; the bottom and top of the box are the 25th and 75th percentiles. Whiskers (errors bars) above and below the box indicate the maximum and minimum value. The correlation between four microRNAs expression and GNMT gene expression level was analyzed using 10 paired HCC specimens from TLCN. T, HCC tumor tissue; TA, tumor-adjacent liver tissue. (**B**) The GNMT and four microRNAs expression levels (left panel) were analyzed using TCGA database. The correlation between four microRNAs expression and GNMT gene expression level was analyzed using 49 paired HCC specimens from TCGA. Reads per million (RPM) mRNA or microRNA mapped values were used to represent microRNA expression levels.*P < 0.05, **P < 0.01, ***P < 0.001, ****P < 0.0001; NS, Not significant, in the comparison between tumor and tumor adjacent liver tissue.

We used both miRWalk^25^ and miRTar^26^ software to identify potential 112 microRNAs targeting at the CDS and 3′UTR of human GNMT mRNA (Supplementary Table S1), while up-regulated 67 miRNAs in HCC were found through literature review^17,27,28^. Initially, we found up-regulation of miR-224, miR-491-5p and miR-93 candidates between software and literature review (Supplementary Fig. S1). The putative binding site of miR-491-5p resides in the 3′UTR of GNMT, while the putative binding sites of both miR-224 and miR-93* are located in the CDS region of GNMT. To explore the relationship between GNMT and these three microRNAs, we analyzed their expression levels in two groups of HCC patients. Consistent with previous reports^17,27,28^, the expression levels of miR-224, miR-491 and miR-93* were up-regulated in HCC while GNMT was down-regulated in tumor tissues from 10 HCC patients (Fig. 1A, left panel). The expression level of GNMT mRNA was inversely correlated with both miR-224 and miR-93* (R = −0.624, R = −0.457) (Fig. 1A right panel). A similar relationship was found in another group of HCC patients consisting of 371 HCC tumor tissues and 49 non-tumorous tissues from TCGA (Fig. 1B). Among these candidates, miR-224 showed the highest significance in upregulated expression and an inverse correlation with the gene expression of GNMT in HCC tissues, we decided to further investigate its role in the regulation of GNMT gene expression.

### miR-224 directly targets the CDS of GNMT mRNA

**Figure 2:**
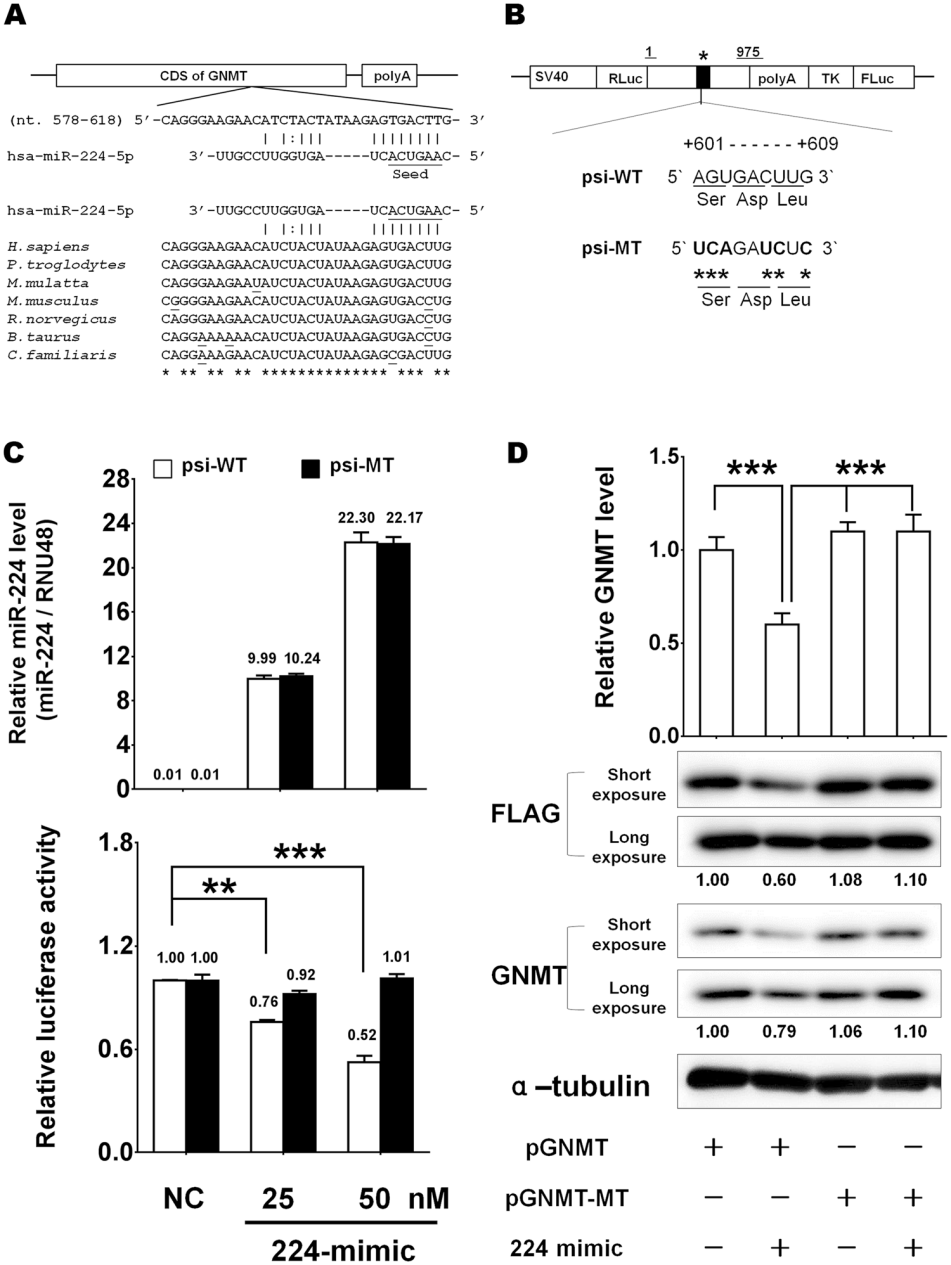
GNMT is a direct target of miR-224. (**A**) GNMT had a putative response element of hsa-miR-224 as predicted by miRWalk and miRTar algorithms. The sequence of miR-224 was aligned with the CDS of GNMT in human (*Homo sapiens*), chimpanzee (*Pan troglodytes*), monkey (*Macaca mulatta*), mouse (*Mus musculus*), rat (*Rattus norvegicus*), cow (*Bos taurus*) and dog (*Canis familiaris*). (**B**) Schematic representation of dual luciferase reporter plasmids containing wild type (psiCHECK2-GNMT-WT, psi-WT) and mutant GNMT (psiCHECK2-GNMT-MT, psi-MT). The asterisk indicates the position of the putative miR-224 binding site, which was mutated in psi-MT reporter plasmid. (**C**) HEK293T cells were transfected with psi-WT or psi-MT plasmid and co-transfected with 224-mimic or NC, respectively. The miR-224 expression level of each sample was normalized with the level of RNU48. The Renilla luciferase activity was determined and normalized with firefly luciferase activity. Quantification of the data was shown as mean ± SD (n = 3). (**D**) The mRNA levels of GNMT in HEK293T cells co-transfected with the indicated plasmids and 50 nM 224-mimic were measured by real-time PCR and normalized with control group (upper panel). **P < 0.01; ***P < 0.001. Whole cell extracts of the transfected cells were harvested for western blot analysis (bottom panel). The normalized GNMT-Flag and GNMT level are presented below each lane.

The nucleotides 601–609 in the CDS of GNMT mRNA serve as a miR-224 response element. The nucleotide sequence of this putative response element is highly conserved in the GNMT gene of human, chimpanzee, monkey, mouse, rat, cow and dog species, suggesting that the sequence is retained through evolution (Fig. 2A). We first verified the authenticity of the CDS binding site using a 3′UTR reporter assay. Both wild type and mutant GNMT CDS containing a mutated miR-224-binding site were subcloned into a 3′UTR dual-luciferase reporter vector and designated as psi-WT and psi-MT, respectively (Fig. 2B). Subsequently, we measured luciferase activity of these two constructs after they were co-transfected with hsa-miR-224 mimic (224-mimic) or negative control (NC). The results showed that the luciferase activity of psi-WT/224-mimic was down-regulated significantly compared to that of psi-WT/NC (P < 0.01, Fig. 2C). Furthermore, no significant difference in luciferase activity was detected between psi-MT/224-mimic group and psi-MT/NC group (Fig. 2C). Therefore, the miR-224 binding site resides in the nucleotides 601–609 of the CDS of GNMT. We then investigated whether miR-224 influenced exogenous GNMT expression. We performed western blot analysis of GNMT in HEK293T cells transfected with a GNMT-FLAG fusion construct (pGNMT plasmid). Transient transfection of pGNMT led to high expression of GNMT (Fig. 2D). HEK293T cells were further cotransfected with 224-mimic (pGNMT/224-mimic) or 224-inhibitor (pGNMT/224-inhibitor). Two known miR-224 target genes, API-5 and SMAD4, served as positive controls^19,23^. The RT-qPCR and western blot assays showed that both mRNA and protein levels of exogenous GNMT were markedly suppressed in the pGNMT/224-mimic group compared to that of pGNMT group (Supplementary Fig. S2). Moreover, 224-inhibitor (pGNMT/224-mimic/224-inhibitor group) rescued both gene expression and protein expression of GNMT that were repressed by 224-mimic. These results confirmed that miR-224 regulates exogenous GNMT expression through interaction with its protein coding region *in vivo*. Additionally, we generated a CDS-only GNMT-MT-FLAG construct containing the miR-224 binding site silent mutation (pGNMT-MT). Transit transfection of pGNMT and pGNMT-MT along with 224-mimic showed that both gene expression (Fig. 2D, upper panel) and protein levels (Fig. 2D, lower panel) were significantly reduced in pGNMT/224-mimic group but not in pGNMT-MT/224-mimic group. Altogether, our current findings unequivocally confirm that miR-224 targets CDS of GNMT mRNA and decreases the mRNA and protein levels of GNMT.

### GNMT and miR-224 have inverse relationship of gene expression in both liver cancer cell lines and tumor tissues

**Figure 3:**
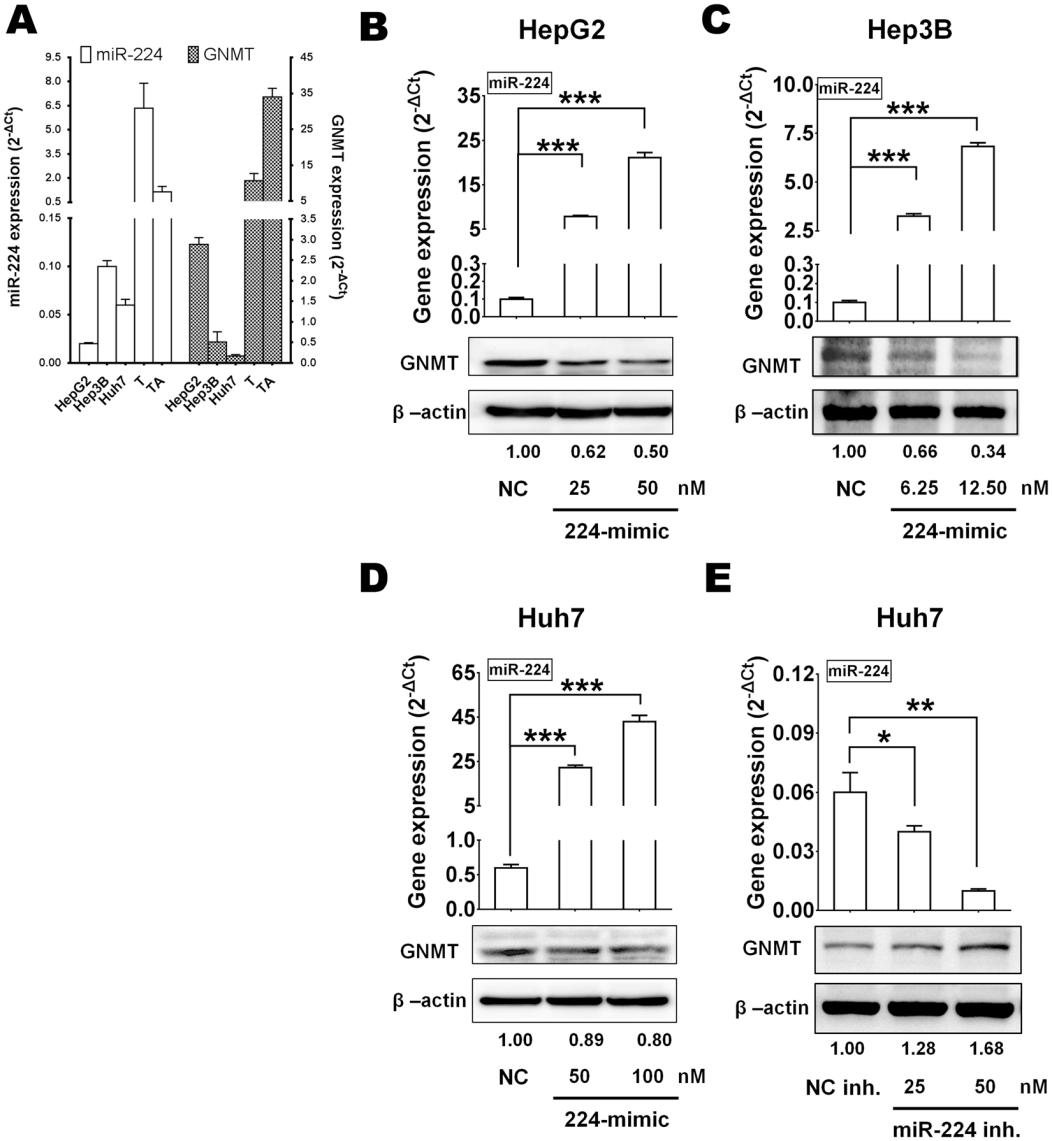
GNMT was suppressed by miR-224 in liver cancer cell lines. (**A**) The expression levels of miR-224 and GNMT in three liver cancer cell lines and 78 pairs of HCC patients were measured by RT-qPCR. T, HCC tissue, TA, tumor-adjacent liver tissue. The expression levels of miR-224 and GNMT were measured in HCC cell lines transfected with (**B**–**D**) 224-mimic and (**E**) 224-inhibitor, control group NC or NC inhibitor by qPCR (upper panel) and western blotting (bottom panel). The GNMT and miR-224 expression levels of each sample were normalized with the level of TBP and RNU48. β-actin was used as internal control for western blotting. *P < 0.05, **P < 0.01, ***P < 0.001.

**Figure 4:**
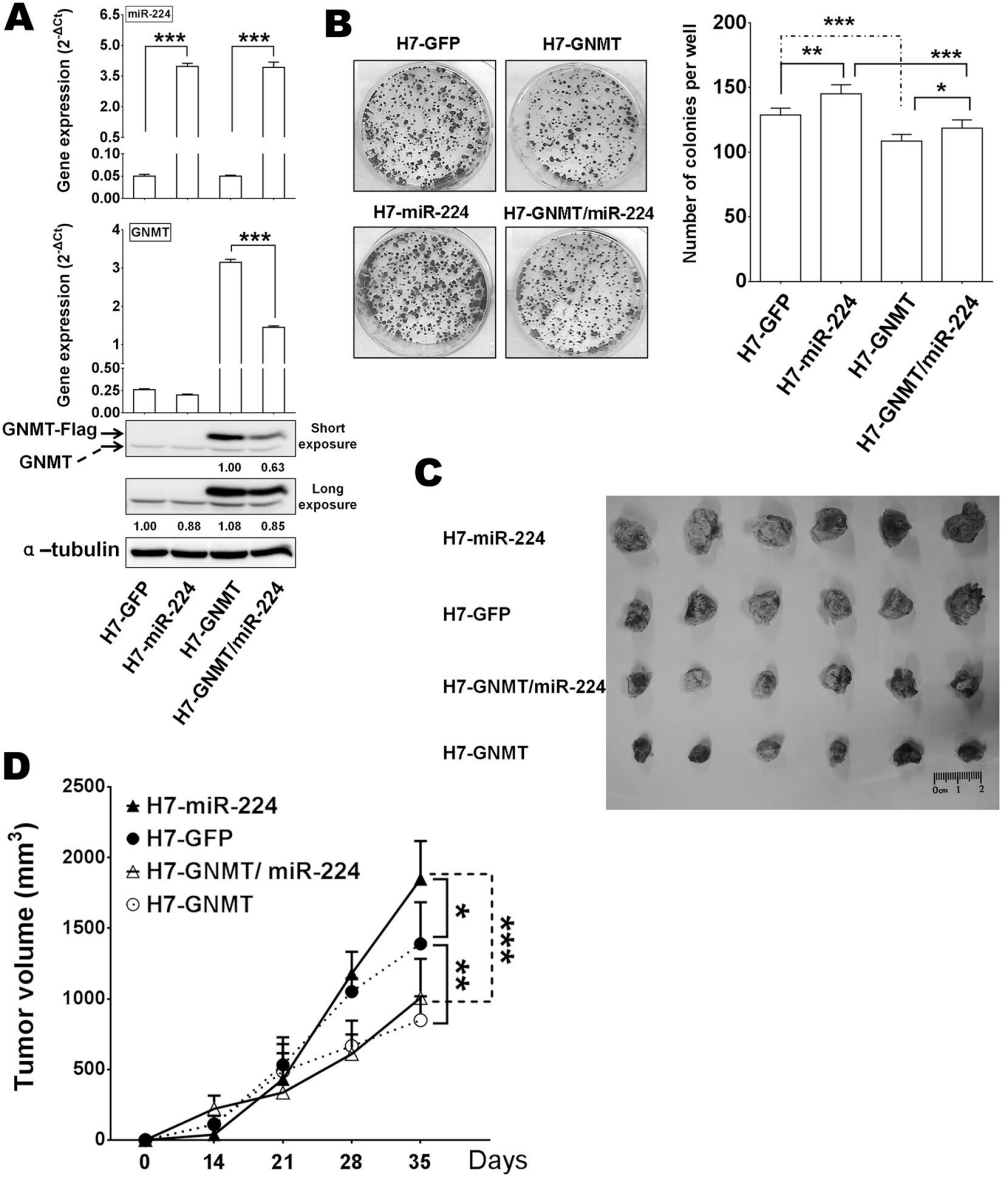
miR-224 induced tumor growth of HCC cells through regulation of GNMT. The effect of lentivirus expressing miR-224 and GNMT on Huh7 cells growth *in vitro* and *in vivo*. (**A**) The GNMT, miR-224 and GNMT/miR224 transcript levels in Huh7 infected with lentivirus expressing miR-224 and GNMT were measured by qPCR (upper panel). (**B**) The colony formation was quantified by staining crystal violet. (**C**) A subcutaneous xenograft of lentivirus expressing eGFP, GNMT, miR-224 and GNMT/miR-224 of Huh7 was injected into NOD/SCID mice (N = 6, per group). The length of formed tumors was recorded in centimeters. (**D**) The tumor growth curve was measured every 7 days for 35 days after inoculation. GNMT protein expression examined by western blot analysis using anti-GNMT and ant-α-tubulin antibodies (bottom panel). *P < 0.05, **P < 0.01, ***P < 0.001.

To investigate whether miR-224 regulated endogenous GNMT in liver cancer cell lines, the expression levels of miR-224 and GNMT in HepG2, Hep3B or Huh7 cell lines were measured by RT-qPCR. As shown in Fig. 3A, Hep3B and Huh7 express higher level of miR-224 and low GNMT, while HepG2 expresses relatively higher level of GNMT. A distinct inverse gene expression pattern of GNMT and miR-224 was evident in the tumor tissues of 78 pair of HCC samples than in the tumor adjacent tissues (Fig. 3A). A dose-dependent repression of GNMT protein expression was detected in all three liver cancer cell lines transfected with 224-mimic (Fig. 3B–D). In addition, the miR-224 inhibitor increased GNMT protein level in Huh7 cells in a dose-dependent manner (Fig. 3E). The fact that endogenous GNMT was regulated in the presence of miR-224 suggests that miR-224 might be a physiological relevant regulator of GNMT expression. miR-224 regulation of endogenous and exogenous GNMT was also detected in Huh7 and HepG2 cells stably overexpressing miR-224 (Fig. 4A and Supplementary Fig. S3A). The results showed that overexpression of miR-224 significantly decreased GNMT protein level in these stably infected cells.

### miR-224 enhances hepatoma cell proliferation *in vitro* and tumorigenicity *in vivo* through regulation GNMT

To test whether ectopic miR-224 overexpression enhanced cell proliferation through regulation of GNMT, we examined the proliferation of HepG2 in alamar blue proliferation assay and found that the miR-224-expressing (G2-miR-224 cell) group exhibited a greater proliferation potential than the G2-GFP (control lentivirus-infected cells, eGFP-expressing), G2-GNMT (GNMT-expressing) and G2-GNMT/miR-224 (both GNMT and miR-224-expressing) group (Supplementary Fig. S3B). Further evidence of the ability of miR-224 to suppress GNMT was measured by colony formation activity of HepG2 and Huh7 cell *in vitro*. The miR-224 cell group formed more colonies than that of the GFP, GNMT and GNMT/miR-224 group (Fig. 4B, Huh7, Supplementary Fig. S3C, HepG2). Moreover, the GNMT/miR-224 group produced elevated or reduced number of colonies when compared with the GNMT group or miR-224 group cells, respectively. GNMT seems to portray an anti-malignancy role in miR-224 expressing cells. To obtain further insight into the biological relevance of miR-224 down-regulating GNMT, we monitored its effect on the tumorigenic properties of Huh7 cells in a xenograft model. Huh7 cells over expressing eGFP, GNMT, miR-224 or GNMT and miR-224 were inoculated into NOD/SCID mice. Consistent with the *in vitro* findings, the enhanced tumor sizes by miR-224 were attenuated by GNMT expression in the GNMT/miR-224 group. The mean tumor volume of the GNMT/miR-224 group was significantly reduced in comparison to that of the miR-224 group (Fig. 4C). The volumes and weights of the tumors derived from the H7-miR-224 group cells were large than that of other stable cell lines (Fig. 4D and Supplementary Table S2). The cell growth level was measured by cell numbers, the growth rate of miR-244 over-expression cell was significantly higher than eGFP transfected cells, however, GNMT over an expressed cell with lower growth rates, co-expressed miR-224 and GNMT had medium growth ratio, these observations collectively suggest that GNMT overexpression in these stable cells reduced cell proliferation in miR-224 expressing groups (Fig. 4D). Therefore, miR-224 plays a tumor-promoting role in colony formation *in vitro* and tumorigenesis of Huh7 cells *in vivo* by targeting GNMT. These results suggest that miR-224 converts hepatoma cells into a highly malignant form through regulation GNMT.

### Level of GNMT is inversely correlated with miR-224 expression in association with hepatitis B virus associated HCC and HBx-transgenic mice

**Figure 5:**
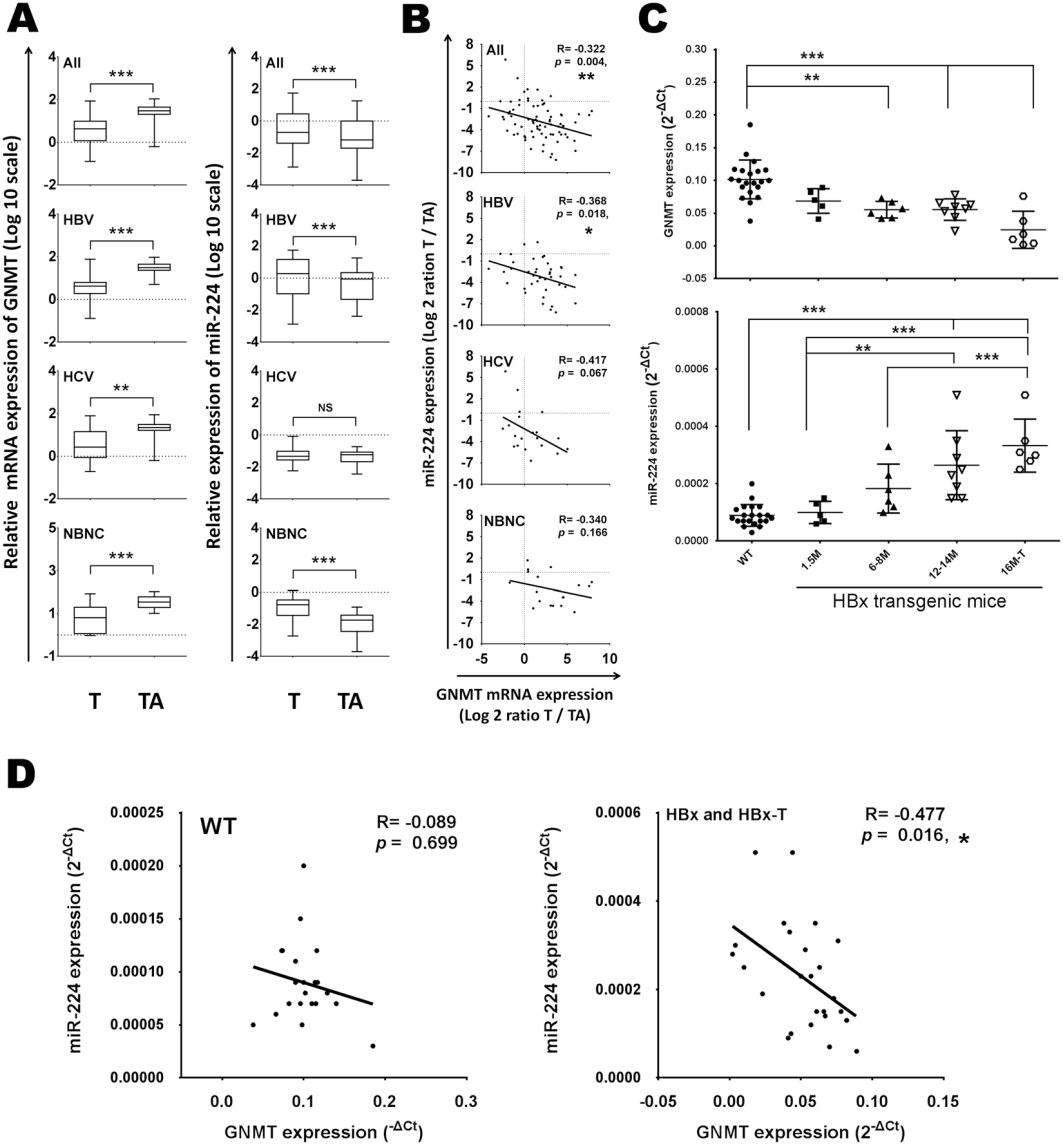
The mRNA expression of GNMT was negatively correlated with miR-224 in HBV-associated HCC specimens and transgenic HBx mice. (**A**) The expression levels of GNMT and miR-224 were measured by RT-qPCR in 40 pairs of HBV-associated HCC, 20 pairs of HCV-associated HCC and 18 pairs of HCC patients without hepatitis viral infection. The relative expression fold of GNMT and miR-224 were normalized to TBP and RNU48 RNA, respectively. (**B**) Pearson correlation analysis for miR-224 and GNMT expressions in 40 paired HBV-associated HCC, 20 paired HCV-associated HCC and 18 paired HCC patients without hepatitis viral infection. T, HCC tissue, TA, tumor-adjacent liver tissue. (**C**) The expression levels of GNMT and miR-224 measured by qPCR in liver without tumor were from 1.5-, 6~8-, 12~14-month-old wild type (WT: 1.5 M, n = 3; 6–8 M, n = 6; 12–14 M, n = 12, total n = 21) and transgenic HBx mice (HBx: 1.5 M, n = 5; 6–8 M, n = 6; 12–14 M,n = 8), and in liver with tumor was from 16-month-old transgenic HBx mice (16M-T, n = 6). The relative expression fold of GNMT and miR-224 were normalized to mouse β-2-microglobulin (β2m) and sno202 RNA, respectively. (**D**) Pearson correlation analysis for GNMT and miR-224 expressions in WT mice liver or HBx mice liver with/without tumor (HBx and HBx-T). *P < 0.05, **P < 0.01, ***P < 0.001; NS, Not significant.

To investigate whether the relationship between GNMT and miR-224 is correlated with viral hepatitis, we extended the analysis to 78 pairs of HCC tumor and tumor-adjacent tissues from TLCN. These clinical samples were categorized into three groups: hepatitis B virus surface antigen (HBs Ag)-positive (HBV group), anti-hepatitis C virus (HCV) antibody positive (HCV group) and negative for both HBs Ag and anti-HCV antibody (NBNC group). Expectedly, the expression of GNMT was significantly lower and that of miR-224 was significantly higher in HCC tumor tissues than in the tumor-adjacent tissues (Fig. 5A). Although a significant increase of miR-224 mean level was detected in All and the groups of HBV and NBNC, a significant inverse correlation between miR-224 and GNMT expression was only seen in All 78 HCC and HBV-related HCC specimens (*R* = −0.322, *P* = 0.004; *R* = −0.368, *P* = 0.018) (Fig. 5B). To further elucidate the role of miR-224 and GNMT in development of liver tumors, the HBx transgenic model was used to confirm the relationship between GNMT and miR-224 expression in HBV-induced hepatocarcinogenesis. Low GNMT and high miR-224 expression were detected in the liver with/without tumors of HBx-transgenic mice (Fig. 5C). A significant inverse correlation between miR-224 and GNMT expression was seen in HBx transgenic mice liver and tumor (*R* = −0.477, *P* = 0.016) (Fig. 5D), which was consistent with the clinical findings in HBV-related HCC specimens. Together, these findings suggest that miR-224 could be involved in the host response to HBV hepatitis.

### GNMT protected against the CCl4-induced miR-224 expression and fibrosis formation in mouse liver

**Figure 6:**
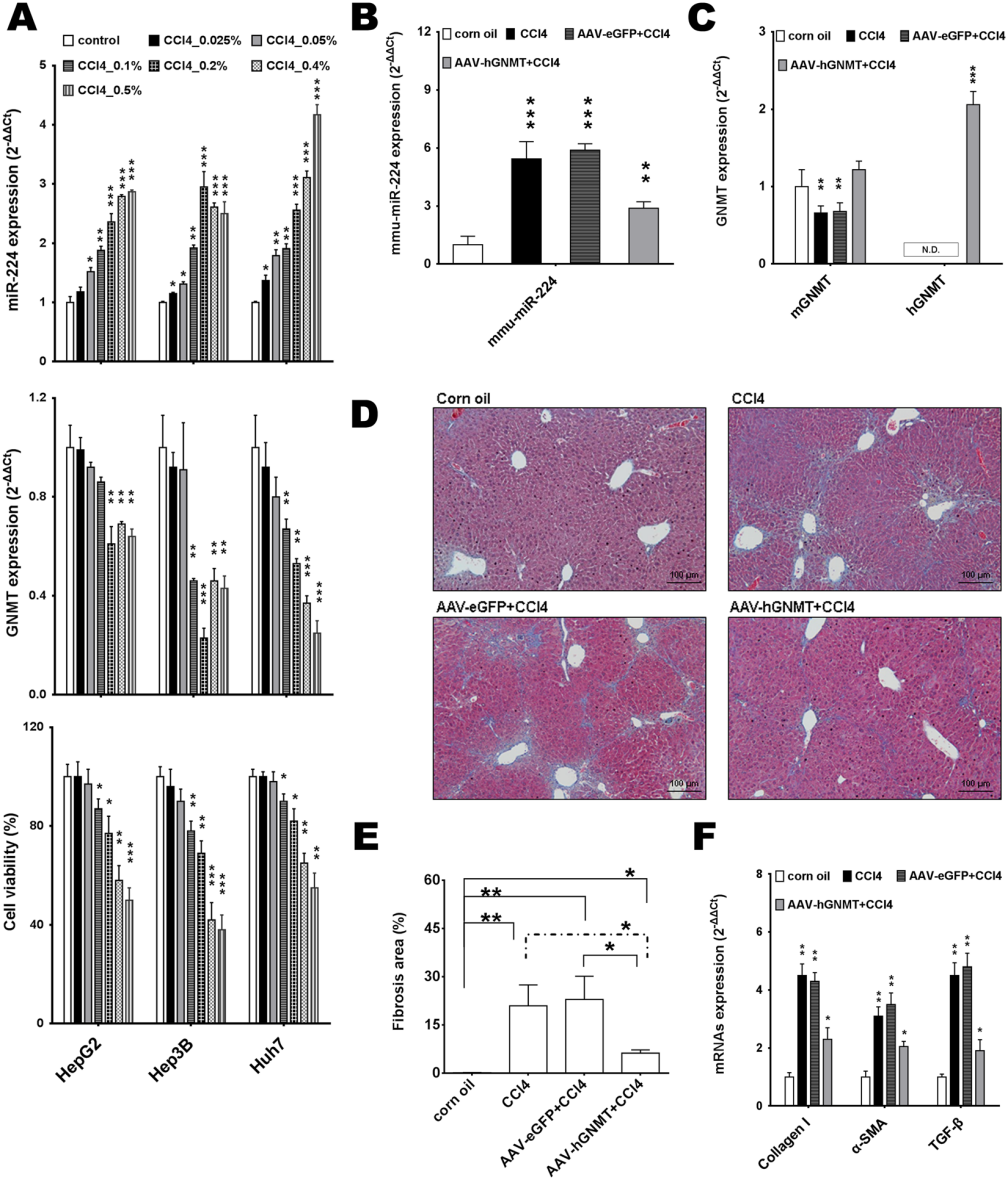
GNMT was related to CCl4-induced miR-224 expression and hepatic fibrosis. (**A**) The expression levels of GNMT and miR-224 were measured by RT-qPCR in CCl4-treated three cell lines at 24^th^ hour. The cell viability (%) was determined by alamarBlue assay (bottom panel). The relative expression fold of GNMT and miR-224 were normalized to cell line of control group. CCl4 exposure induced cell death in three cell lines in a dose dependent manner. The male balb/c mice were treated with 20 mg/kg of CCl4 twice per week for 8 weeks post 2 weeks of 10^11^ copies/mouse of AAV injection. The gene levels of (**B**) mmu-miR-224, (**C**) mouse GNMT (mGNMT) and human GNMT (hGNMT) genes in liver of CCl4-treated mice after AAV injection were measured by RT-qPCR. (**D**) The liver section of experimental mice was examined by Masson’s Trichrome stain. The result of 4 groups including groups of corn oil, CCl4, AAV-eGFP plus CCl4 and AAV-GNMT plus CCl4 are shown. The black bar is representative to length of 100 μm. (**E**) The area of fibrosis (positive Masson’s trichrome staining) was measured by morphometry in the four groups. (**F**) The expression levels of collagen I, α-SMA and TGF-β genes in these four groups were measured by RT-qPCR. The RT-qPCR Data were normalized to expression levels of the human TBP, mouse β2m, RNU48 and sno202 RNA across each treatment. The relative expression fold of genes and miR-224 were normalized to control group, respectively. The data represent the mean ± SD, n = 3. Significant differences are indicated by *P < 0.05, **P < 0.01, ***P < 0.001 as compared to the control group. ND, not detected.

We subsequently examined whether hepatic fibrosis-induced miR-224. To explore the correlation between miR-224 and GNMT expression in CCl4 intoxication, we designated the concentration-dependent cytotoxic effect of CCl4 on HepG2, Hep3B and Huh7 cell lines. The dose-dependent increase of miR-224 expression and decrease of GNMT expression proved the establishment of the CCl4-induced miR-224 and –repressed GNMT model (Fig. 6A–C). The expression of GNMT was significantly lower and that of miR-224 was significantly higher in CCl4-treated liver cancer cell lines and mouse liver tissue than in the control group. Liver cancer cell lines were affected by CCl4 exposure in a dose-dependent manner causing remarkable loss of cell viability (Fig. 6A). In addition, GNMT was regulated by miR-224 in human cells (HEK293T) and mouse cells (Hepa1–6) transiently cotransfected with pGNMT/hsa-miR-224 mimic and pGNMT/mmu-miR-224 mimic (Supplementary Fig. S4). We then examined the function of GNMT in CCl4-induced liver injury in mice, used the adeno-associated virus (AAV)-mediated human GNMT in mouse liver for overexpression, which was followed by CCl4 treatment. RT-qPCR analysis revealed that the expression of miR-224, collagen type I (collagen I, a extracellular matrix protein), α-smooth muscle actin (α-SMA, a typical marker of activated hepatic stellate cells) and transforming growth factor beta 1 (TGF-β1, a major profibrogenic cytokine) were significantly increased and mouse GNMT was significantly decreased by CCl4 treatment (Fig. 6B,C,F). Moreover, the collagen deposition in liver was examined by Masson’s trichrome staining after the 8 weeks of CCl4 injections. The results revealed the most of mice in the AAV-GNMT/CCl4 group exhibited obvious and uniform hepatic fibrosis (Fig. 6D). The severity of the hepatic fibrosis was milder in the AAV–GNMT/CCl4 group than that in either the AAV-eGFP/CCl4 or CCl4 groups (Fig. 6E). Accordingly, miR-224, collagen I, α-SMA and TGF-β1 expression in AAV-GNMT/CCl4 mice was lower than those in the AAV-eGFP/CCl4 and CCl4 groups, whereas expression was significantly higher than in the corn oil group. These results collectively demonstrate that GNMT plays a protective function in CCl4-induced liver injury.

## Discussion

GNMT is a tumor suppressor gene for HCC but its regulatory mechanism is not well understood. In this study, we identified miR-224 as the key regulator of gene expression of GNMT, mapped its seeding site and further created a GNMT cDNA mutant which can resist to the miR-224 regulations. We believe that such mutant GNMT cDNA potentially can serve as gene therapy materials for chronic liver diseases including HCC.

In a mouse model of hypophysectomy operation, the gender disparity of GNMT gene expression is regulated by growth hormone^29^. However, GNMT down-regulation is complex in HCC. In our previous study, we characterized the GNMT core promoter region and xenobiotic responsive elements in HEK 293 A and HepG2 cells and androgen response element in LNCaP cells^30,31^. GNMT promoter activity could be induced by benzo[a]pyrene (BaP) through the xenobiotic responsive element in HEK 293 A by BaP- or R1881 (a synthetic androgen receptor agonist)-induced GNMT mRNA expression in HepG2 or LNCaP cells. Recently, we identified 1,2,3,4,6-penta-O-galloyl-beta-D-glucopyranoside (PGG) as a GNMT promoter enhancer compound in HCC^32^. Moreover, we demonstrated that PGG enhances GNMT promoter activity and mRNA expression via inhibition of c-Myc expression in HCC (unpublished data). These results are valuable in the investigation of the regulatory mechanisms of GNMT gene expression, especially in the pathogenesis of cancer. Furthermore, our study of 5 HCC cell lines and 16 non-paired HCC tissues from Taiwanese patients showed no significant variation (mutations or deletion) in the CDS of GNMT, which suggests that the down-regulation of GNMT in HCC may not be caused by gene mutations^5^. To further understand the potential mechanisms that lead to the down-regulation of GNMT in cancer, previous studies of hypermethylation of the GNMT promoter showed that the 3′ region of the TSS of GNMT contained seven CpG sites which were methylated in HCCs (20%)^33^. The GNMT promoter was found to be tumor-specific and its effect consisted in the down-regulation of gene expression in pancreatic ductal adenocarcinoma (PDAC) samples and in PDAC cell lines^34^. However, demethylating drug (5-aza-2′-deoxycytidine, DAC) treatment did not lead to significant induction of GNMT mRNA (less than 1.5-fold induction) in liver cancer cell lines. Moreover, no significant association between DNA methylation and GNMT mRNA expression was found in HCC^33^. Collectively, promoter hypermethylation does not appear to be a crucial contributor in transcriptional silencing of GNMT in HCC. However, the contribution of these factors in GNMT downregulation is not studied in HCC. Herein, we aimed to investigate the microRNA-guided regulation of GNMT in HCC.

In this study, we found that miR-224 targeted at the CDS of GNMT gene. Although most of miRs target at the 3′UTR, recent studies demonstrated that microRNA can also target at the CDS^35^. Notably, three miR-224, miR-491 and miR-93* have been reported to be up-regulated in HCC^27,36–41^. In addition, RT-qPCR revealed negative relationship between the GNMT mRNA levels and expression of miR-224 and miR-93*, suggesting that downregulation of GNMT in HCC may be due, at least in part, to the overexpression of miR-224 and -93* (Fig. 1). miR-224 is frequently upregulated in HCC and thus known as an onco-miRs for HCC^18,21^. In terms of miR-93*, it was found to be up-regulated significantly in both TLCN and TCGA cohorts (Fig. 1). The hsa-miR-93* is located at the opposite arm of the hsa-miR-93 which has been reported to be up-regulated in HCC. It has been shown that hsa-miR-93 can inhibit apoptosis and it is associated with cellular proliferation, migration and invasion^28^. In this study, we found that GNMT mRNA expression level negatively correlated with miR-93* expression in both TLCN and TCGA cohorts although the p value is insignificant (R = −0.457, p = 0.123 and R = −0.250, p = 0.083, respectively). Further studies are needed to confirm this preliminary finding. Our results showed that the expression level of miR-224 was not significantly inversely correlated with GNMT mRNA in 49 paired HCC specimens from the TCGA cohort. This differed from the results of the analysis of 78 paired HCC specimens from TLCN cohort. The difference may be attributed to the different sample size, ethnicity and environment. However, the level of GNMT was inversely correlated with miR-224 expression in both TLCN and TCGA cohorts. Therefore, the exact mechanisms underlying still remain elusive and need further in-depth study.

The GNMT 3′UTR sequence was rather short which made searching for microRNAs targeting the 3′UTR difficult. Fang and Rajewsky found that microRNA target sites present in both the CDS regions and the 3′UTRs have synergistic effects^42^. Reczko *et al*. used computational model for microRNA target genes and found that genes with shorter 3′UTRs favorably targeted in the CDS suggested that the evolutionary pressure might favor the presence of additional sites on the CDS if there is restricted space on the 3′UTRs^35^. These findings help explain the functional importance of microRNA targeting in coding regions. In order to determine whether silent mutation of the miR-224 binding site in the GNMT CDS reduced side effects by other microRNAs, the minimal side effects of the mutation sequence were analyzed by RegRNA 2.0 (an integrated web server for identifying functional RNA motifs and sites) and miRbase database. We found that the silent mutation sequence (UCAGAUCUC) acted as an escape of miR-224 repression. Thus, GNMT-MT (silent mutant type) is a potential therapeutic approach to recover GNMT expression attenuating liver injury in HCC.

Furthermore, upregulation of miR-224 is reported to be involved in HBV- or HCV-associated HCC^23,43–45^. Our study showed that miR-224 was upregulated in HCC, especially among HBV-related HCC and HBV/HCV negative HCC patients (Fig. 5A). The limitation of the HCV-associated HCC sample set is relatively small sample size. However, the mechanisms by which miR-224 are upregulated in HCCs have not been fully elucidated. Interestingly, miR-224 was regulated by HDAC1, HDAC3, EP300 and NF-*κ*B in HCC cells^46,47^. NF-*κ*B-dependent inflammatory pathways were induced by chronic hepatitis B or C viral infections in HCC development^48^. HBV-associated chromatin modifying enzymes of PCAF, p300/CBP, HDAC1, SIRT1, and EZH2 have been shown to bind to the cccDNA in human hepatoma cells containing replicating HBV^49^. Furthermore, Lan and colleagues reported that a mechanism of autophagy in the miR-224-Smad4 pathway was related to tumorigenesis in liver cancer. They found that reduction of autophagy correlated with high miR-224 expression was only displayed in HBV-related HCC specimens for virus-associated HCC, which was confirmed in an HBx-transgenic mice model (Fig. 5C). Notably, miR-224 expression was found to be moderately elevated in chronic hepatitis and liver cirrhosis^37^. Compared to normal liver samples, expression was higher in nontumor tissue of patients with HCC and highest in tumor of patients with HCC^46^. Thus, these findings suggest that inflammation-induced miR-224 expression may contribute to HCC development.

Chronic HBV infection and environmental exposure to environmental toxins such as aflatoxin B_1_ (AFB_1_), aristolochic acid (AA), CCl4 and N-nitrosodiethylamine (DEN) are risk factors that contribute to the development of chronic liver failure resulting in liver cirrhosis or HCC. In addition, CCl4 enhance hepatocarcinogenesis in AFB_1_ or DEN-induced animal model of fibrosis and inflammation-associated HCC^50,51^. CCl4, a potent hepatotoxic chemical, is extensively used to induce acute liver injury in experimental animals^52^. Toxicity of CCl4 was the main cause of CCl4-induced oxidative stress and inflammation *in vitro* and *in vivo*^53^. However, the TaqMan Array rodent 155 microRNA platform for DEN/CCl4-induced fibrosis-associated liver carcinogenesis in mice did not include mmu-miR-224^54^. In contrast, we found that miR-224 induced in CCl4-treated liver cancer cell lines and CCl4-treated mice (Fig. 6). Furthermore, it has been shown that hsa-miR-224 expression induced in acetaminophen-induced hepatotoxicity^55^. Thus, environmental toxins-induce miR-224 expression during liver injury. GNMT is involved in the cellular mTOR pathway and cholesterol homeostasis^3^. In addition, hepatic inflammatory infiltration as well as inflammatory signals (IL-6, TNF-α and IL1-β) were up-regulated and altered hepatic detoxification and anti-oxidation facility, which may accumulate lipid peroxidation and result in liver damage in Gnmt−/− mice^56,57^. We demonstrated that hepatic GNMT regulated lipid and glucose homeostasis, and provided insight into the development of insulin resistance by modulating the PI3K/Akt pathway^56^. Furthermore, GNMT was expressed abundantly in mouse liver tissue but diminished tumor tissues from AFB1-treated wild-type mice^58^. Therefore, GNMT plays a crucial role in the pathological process of different liver diseases including chronic hepatitis, glycogen storage, hypercholesterolemia, fatty nodules, and liver cancer. Recently, high rates of AA and their derivatives have been implicated in the mutational spectra in Taiwan HCC^59^. We also found GNMT down-regulation and miR-224 up-expression in the liver of AA-treated mice after 3-week AA exposure^60^. AA may decrease GNMT expression by increasing miR-224. Therefore, we focused on the relationship between GNMT and miR-224 in CCl4-induced liver injury and HBx transgenic mouse model. Interestingly, we also observed a reduction in the expression of GNMT and a significant inverse correlation between miR-224 and GNMT expression in CCl4-induced inflammation response and oxidative stress of liver cancer cell lines and mice (Fig. 6A–C). HBV-related HCC specimens (Fig. 5C), HBx transgenic mouse model and (Fig. 5D) and miR-224 overexpression enhanced and GNMT overexpression reduced tumor growth *in vivo*, which is consistent with the *in vitro* data (Fig. 4 and Supplementary Fig. S3), In addition, AAV-GNMT reduced CCl4-induced miR-224 expression and fibrosis formation in mouse liver (Fig. 6B,C,F). Therefore, we propose that miR-224/GNMT may act as a bridge between inflammation and tumorigenesis (Supplementary Fig. S5). Further investigation is required to delineate the interaction between HBV infection and the regulation of miR-224. Moreover, GNMT participates in this interaction also needs to be elucidated.

In conclusion, our study demonstrated that miR-224 is involved in the down-regulation of GNMT in HCC. Furthermore, our data indicates that miR-224 plays an important role in tumorigenesis of human liver cancer cells by directly suppressing the GNMT tumor suppressor. This study provides a new therapeutic strategy of HCC by manipulating the level of GNMT using miR-224 inhibitors. Alternatively, silent mutants of GNMT cDNA which can resist miR-224′s attack can be used in gene therapy. Nevertheless, miR-224 cannot completely inhibit GNMT in these liver cancer cell lines suggesting that the downregulation of GNMT is very complex mechanism in hepatoma cells (Figs 3, 4A and Supplementary Fig. S3A). Therefore, further investigations on the regulatory mechanisms of GNMT are needed and should provide new insights into hepatocarcinogenesis.

## Materials and Methods

### Specimens from HCC patients

RNA specimens were obtained from the following two groups of patients through the Taiwan Liver Cancer Network (TLCN, http://140.112.133.121/index.action): tumor and tumor-adjacent tissue pairs from 78 HCC patients (Supplementary Table S3). Five medical centers; National Taiwan University Hospital, Chang-Gung Memorial Hospitals at Linko and Kaohsiung, and Veterans General Hospitals at Taichung and Kaohsiung, participate in the TLCN. Preoperative written informed consents were obtained from all the patients recruited by the TLCN. These 78 HCC patients were subcategorized into three groups: 40 patients (20 males and 20 females) were HBsAg-positive, 20 patients (10 males and 10 females) were anti-HCV Ab-positive and 18 patients (9 males and 9 females) were negative for both HBsAg and anti-HCV antibody. The study protocol has been approved by the Institutional Review Board of the National Yang Ming University (IRB No. 1000041) and the user committee of the TLCN (No.070001 and No.120060). The clinicopathological profiles of the patients enrolled in the study are shown in Supplementary Table S3.

### Identification of the microRNAs that target at GNMT mRNA

We used both miRWalk^25^ and miRTar^26^ tools to identify microRNA targeting sites in human GNMT mRNA. All the microRNAs that potentially target at 3′UTR and CDS of GNMT mRNA were selected. Next, we picked up those microRNAs associated with HCC through literature review and database query using both TarBase^61^ and miRTarBase^62^. Besides, we analyzed the conserved regions of microRNA target sites among several mammalian species including human chimpanzee, monkey, mouse, rat, cow and dog.

#### Expression datasets

Human HCC microRNA and mRNA expression datasets were retrieved from The Cancer Genome Atlas (TCGA, https://tcga-data.nci.nih.gov/tcga) of Liver hepatocellular carcinoma (LIHC). To provide the unique data types, we have only collected the newest platforms for further analysis (microRNA-seq from Illumina HiSeq and RNA-seq from version2 Illumina HiSeq). Finally, we collect 420 samples containing 371 tumors and 49 tumor adjacent tissues.

### Cell lines and cell culture

Human embryonic kidney cell line HEK-293T and Human liver cancer cell lines derived from hepatocellular carcinoma (Huh7 and Hep 3B) or hepatoblastoma (HepG2) were cultured in complete Dulbecco’s Modified Eagle’s Medium (DMEM). DMEM contained 10% heat-inactivated fetal bovine serum (FBS), penicillin (100 U/ml), streptomycin (100 lg/ml), nonessential amino acids (0.1 mM) and L-glutamine (2 mM) (Thermo Fisher Scientific). Cells were transfected into 6 well plates (5 × 10^5^ cells per well) with 6.25~100 nM 224-mimic, and NC, 25~50 nM hsa-miR-224 inhibitor (224-inhibitor) and hairpin inhibitor-negative control (NC inhibitor) (Dharmacon) for microRNA transfection. Transfection was performed using the Trans IT-TKO (Mirus). Plasmid DNA was transfected by using TurboFect (Fermentas). All transfections were performed according to the manufacturer’s instructions.

### Western blot

Cultured cells were lysed by using lysis buffer supplemented with protease and phosphatase inhibitors^9^. Cellular proteins (20 or 100 μg) were separated by SDS-PAGE. The following Ab were used at dilutions recommended by manufacturers: anti-GNMT Ab (YMAC Biotech Co, Taipei, Taiwan), anti-FLAG Ab, anti-β-actin and anti-α-tubulin Ab (Sigma). Immunoblotting signals were normalized to anti-β-actin or anti-α-tubulin and quantified by densitometric scanning.

### Ectopic expression of miR-224

Four lentiviral constructs were made to generate HepG2 and HuH-7 stable cells expressing GNMT, GFP, hsa-miR-224 and control scramble (scrambled miR-224). Two plasmids encoding hsa-miR-224 and control scramble for lentivirus construction (LentimiRa-hsa-miR-224-5p Vector and LentimiRa-scrambled-h224 Vector) (ABM Inc., Canada) were purchased from ABM Incorporation. The pLKO_AS3w.eGFP.puro was obtained from the National RNAi Core Facility (Academia Sinica, Taiwan). Full length GNMT and eGFP were subcloned into pLKO_AS3w.hyg (hygromycin-B phophotransferase), hsa-miR-224 and control scramble subcloned into pLKO_AS3w. puro (puromycin-N-acetyl-transferase) lentiviral expression vector. HEK293T cells were cotransfected with a packaging plasmid-pCMV-Δ R8.91, a VSV-G envelope expressing plasmid-pMD.G and one of the following lentiviral constructs: pLKO_AS3w.eGFP.hyg, pLV-GNMT-FLAG-hyg, pLV-miR-224-puro and pLV-scrambled-h224-puro. A supernatant containing lentiviruses was harvested according to the protocol published on the website http://rnai.genmed.sinica.edu.tw. To generate stable cell lines, they were infected with pseudo-typed lentivirus in medium containing polybrene (8 μg/mL).Twenty-four hours after infection, the cells were treated with puromycin (1 μg/mL) and/or hygromycin (50 μg/mL) to select stable cells. Lentivirus-infected cells including (i) Huh7-eGFP & scramble (H7-GFP), Huh7-GNMT & scramble (H7-GNMT), Huh7-miR-224 & eGFP (H7-miR-224), Huh7-GNMT & miR-224 (H7-GNMT/miR-224); (ii) HepG2-eGFP & scramble (G2-GFP), HepG2-GNMT & scramble (G2-GNMT), HepG2-miR-224 & eGFP (G2-miR-224), HepG2-GNMT & miR-224 (G2-GNMT/miR-224) were grown in DMEM supplemented with puromycin and hygromycin.

### Colony formation assay

Cells were seeded in six-well plates (3,000 cells per well). After 2 weeks, colonies were stained with 0.1% crystal violetin 10% formalin, and the number and area of colonies were analyzed by CellProfiler^11^.

### Xenografted tumorigenicity assay

NOD/SCID mice (4 weeks of age) were purchased from the Laboratory Animal Center of the National Taiwan University College of Medicine, and were injected subcutaneously in the right flank with 1 × 10^6^ of Huh7 stable cell lines. By this time, the HCC xenografts reached the size of approximately 100 mm^3^. Tumor growth was monitored at least twice weekly by Vernier caliper measurement of the length (*L*) and width (*W*) of tumor. Tumor volume (TV) was calculated as follows: TV = (*L* × *W*^2^)/2. Tumor size was measured on day 14, 21, 28 and 35 post injections, and tumor weight was measured on day 35 when the mice were sacrificed. Data are presented as mean of tumor volume ± SEM. The protocol was reviewed and approved by the Institutional Animal Care and Use Committee of Kaohsiung Medical University in compliance to the guidelines on the care and use of animals for scientific purposes.

### Construction of pAAV-GNMT

The construction of the pAAV-eGFP, kindly provided by Dr. Mi-Hua Tao^63^, The construction of the pAAV-eGFP, kindly provided by Dr. Mi-Hua Tao, containing the CMV promoter and the eGFP coding sequence (CDS), has been reported previously. The human GNMT CDS fragment with Flag sequence in C-terminus of GNMT gene was generated by PCR to replace eGFP CDS in pAAV backbone by EcoRI and Asc I. The vectors of AAV-eGFP and AAV-GNMT were prepared by using the AAV Helper-Free System as previously described. Briefly, pAAV-eGFP and pAAV-hGNMT were cotransfected with pHelper and pXX8, respectively, into 293 cells by a calcium phosphate-based protocol. The cells were collected 48 hours later, and the viral particles were harvested. After purification by CsCl gradient centrifugation, the viral particle titers were determined by quantitative real-time PCR and these vectors were applied in the following animal experiments.

### CCl4-induced hepatic inflammation in liver cancer cell lines and mice

Carbon tetrachloride (CCl4) was purchased from Sigma-Aldrich Chemicals Co. HepG2, Hep3B and Huh7 cells were treated with 0.025, 0.05, 0.1, 0.2, 0.4 and 0.5% CCl4 in DMEM culture medium and incubated for 1 h. Cells incubated with fresh medium served as controls. After incubation, the medium was replaced with 4 ml of fresh medium. For GNMT protects mouse liver against CCl4-induced hepatic inflammation and fibrosis experiment, 2-monthold male balb/c mice were used to verify the activity of adeno-associated virus (AAV)-mediated expression of GNMT in liver. 24 mice were divided into four groups: normal controls (treated with corn oil, n = 6), AAV-GNMT/CCl4 (n = 6), AAV-eGFP/CCl4 (n = 6), and CCl4 (n = 6). These groups of mice were given AAV-GNMT (1 × 10^11^ viral genomes in 100ul saline), AAV-eGFP (1 × 10^11^ viral genomes in 100ul saline) or the equal volume of saline by intravenous injection. After 2 weeks of AAV injection, these groups of mice were treated intraperitoneally with CCl4 (1 ml/kg BW dissolved in coin oil; final concentration of 20%) twice a week for 8 weeks. The mice in normal controls were given an equal volume of corn oil. Blood samples were collected 2 days after CCl4 injection every two weeks. The pathological changes in these four groups were monitored by ultrasound imaging. The mice were sacrificed after 8 weeks of CCl4 treatment.

### Statistical analysis

Data are the mean ± SD. Comparisons of mean values between groups were evaluated by two-tailed nonparametric Wilcoxon signed-rank test or Mann-Whitney test. A value of *P* < 0.05 was considered to be statistically significant. Pearson correlation was used to analyze the relationship between the expression of GNMT and microRNAs in HCC patient samples.

## Electronic supplementary material

Supporting information
