# Supplementary material for: MicroRNA-224 down-regulates Glycine N-methyltransferase gene expression in Hepatocellular Carcinoma
Source: Sci Rep. 2018 Aug 16;8:12284. doi: 10.1038/s41598-018-30682-5 (PMC6095880; doi:10.1038/s41598-018-30682-5)
Supplement: Supplementary file 1 — Supporting information [file 41598_2018_30682_MOESM1_ESM.pdf]

MicroRNA-224 down-regulates Glycine *N*-methyltransferase gene expression in Hepatocellular Carcinoma

Jung-Hsien Hung[1,2], Chung-Hsien Li[2], Ching-Hua Yeh[2], Pin-Chen Huang[2], Cheng-Chieh Fang[2], Yen-Fu Chen[2], Kuo-Jui Lee[2], Chih-Hung Chou[3], Hsin-Yun Cheng[2], Hsien-Da Huang[3,4], Marcelo Chen[5,6,7], Ting-Fen Tsai[8], Anya Maan-Yuh Lin[1,9,10], Chia-Hung Yen[2,11], Ann-Ping Tsou[12], Yu-Chang Tyan[2] and Yi-Ming Arthur Chen[2,13]

Supporting Information Table of Contents

Supporting Information Materials and Methods
Supporting Information Tables S1-4
Supporting Information Figure S1-6
Supporting Information Original Picture for Western Blot

Supporting Information

Reverse-transcription and real-time polymerase chain reaction (RT-qPCR). Total RNA was extracted using Trizol reagent (Invitrogen, Carlsbad, CA). RNA was reverse-transcribed using Tetro cDNA Synthesis Kit (Bioline, Tauton, MA) according to the manufacturer's instructions. KAPA SYBR FAST cursor Kits (Kapa Biosystems, Woburn, MA) were used for real-time PCR applications. PCR conditions were as follows: 5 min at 95°C followed by 40 cycles of 95°C for 10 sec, 60°C for 30 sec and 72°C for 30 sec. Primer sequences are shown in Table S4. For the detection of hsa-miR-224, 20 ng of total RNA was reversely transcribed into complementary DNA using TaqMan MicroRNA Assay hsa-miR-224 reverse transcription primer and TaqMan miRNA reverse-transcriptase kit using the instructions provided by the manufacturer (Invitrogen, Carlsbad, CA). The miRNA expression was normalized to the level of RNU48 RNA.

Construction of pGNMT-MT plasmid and 3' UTR reporter assay

To construct plasmid pGNMT-MT containing the cytomegalovirus (CMV) promoter, a FLAG fragment and a miR-224 binding site silent mutation (MT) of full length GNMT coding sequences for FLAG-tagged GNMT, we used pFLAG-CMV-5 (Simga) as a vector and the pGNMT (wild type of GNMT cDNA) plasmid [1] as the PCR template for generating the insert. Mutations of the GNMT cDNA were made using PCR with the QuikChange II (Stratagene) site-directed mutagenesis kit. The presence of correct mutations was confirmed by DNA sequencing. The wild type (WT) and silent mutant (MT) cDNA fragment were subcloned downstream of renilla luciferase gene in a vector psi-CHECK2 (Promega). Detailed procedure of the constructions of the plasmids is illustrated in Figure S6 and primer sequences are shown in Table S4. HEK293T cells were transiently co-transfected with plasmid DNA from psiCHECK2-GNMT-WT (psi-WT) or a binding site mutant plasmid psiCHECK2-GNMT-MT (psi-MT) along with miRIDIAN mimic-negative control (NC) or hsa-miR-224 mimic (224-mimic) (Dharmacon) using TurboFect (Fermentas) and Trans IT-TKO (Mirus). After 48 hours, luciferase activity was measured using the Dual-Luciferase Reporter Assay System Kit (Promega) and Infinite 200 (TECAN) following the manufacturer's instructions. In the assay, renilla luciferase activities were normalized to firefly luciferase activities.

Cell proliferation

Cell proliferation was determined using the alamarBlue assay (Invitrogen, Carlsbad, CA). Ten thousands cells were seeded in triplicates on 24 well plates for 1, 3, 5, 7 and 9days. 100 μl of alamarBlue solution (final concentration of 10% in medium) and cells were further incubated for 4 h at 37°C for a specified time period. After incubation, 100 μl of the alamarBlue solution from each well of the assay plates was transferred to a new well in 96-well plate, then fluorescence was measured at 530/590 nm. Proliferation was compared to that of the 1st day group.

Hepatitis B Virus X Protein (HBx) Transgenic Mice Liver tissues.

HBx transgenic mice (C57B/6 background) were generated as described[2]. Mice Liver tissues were obtained from professor Tsai's group in the National Yang-Ming University. The liver tissues were collected separately from 1.5-, 6-, 12, 16-month-old mice in two different groups: HBx transgenic type and wild-type male (n=3~6). Total RNA was isolated from mouse liver using Trizol Reagent (Invitrogen, Carlsbad, CA).

Masson's Trichrome stain

The collagen deposition in liver tissue was evaluated by Masson's trichrome staining (HT15-1KT, SIGMA-AlDRICH). Brief description: deparaffinize tissue sections and hydrate to deionized water. Stain in working Weigert's Iron Hematoxylin solution for 1 minute. Wash in running tap water for 5 minutes. Rinse in deionized water. Stain in Biebrich Scarlet Acid Fuchsin solution for 10 seconds. Rinse in deionized water. Place slides in working Phosphotungstic-Phosphomolybdic Acid solution for 5 minutes. Stain in Aniline Blue solution for 5 minutes. Rinse in deionized water. Place slides in 1% Acetic Acid solution for 2 minutes. Rinse in deionized water and dehydrate through alcohol 1 min, clear in xylene for 1 minute.

References

1 Chen, S. Y. *et al.* Glycine N-methyltransferase tumor susceptibility gene in the benzo(a)pyrene-detoxification pathway. *Cancer research* **64**, 3617-3623, doi:10.1158/0008-5472.CAN-03-3726 (2004).

2 Wu, B. K. *et al.* Blocking of G1/S transition and cell death in the regenerating liver of Hepatitis B virus X protein transgenic mice. *Biochemical and biophysical research communications* **340**, 916-928, doi:10.1016/j.bbrc.2005.12.089 (2006).

Table S1 The putative microRNAs response elements in the CDS and 3' UTR GNMT mRNA from miRWalk and miRTar software

MicroRNA	Seed Length	Start	Position	End	Region
hsa-let-7f-2*	7	109	2	103	CDS
hsa-miR-1	8	857	2	850	CDS
hsa-miR-1178	8	473	2	466	CDS
hsa-miR-1225-5p	7	748	1	742	CDS
hsa-miR-1226	7	234	2	228	CDS
hsa-miR-1227	7	209	2	203	CDS
hsa-miR-1233	7	60	2	54	CDS
hsa-miR-1236	8	241	1	234	CDS
hsa-miR-1247	7	81	2	75	CDS
hsa-miR-1250	7	476	2	470	CDS
hsa-miR-1265	11	557	2	547	CDS
hsa-miR-127-5p	7	248	1	242	CDS
hsa-miR-1281	7	87	2	81	CDS
hsa-miR-1289	9	223	1	215	CDS
hsa-miR-129*	7	245	1	239	CDS
hsa-miR-129*	7	59	2	53	CDS
hsa-miR-1307	8	139	1	132	CDS
hsa-miR-1321	7	39	1	33	CDS
hsa-miR-1324	8	757	1	750	CDS
hsa-miR-135b*	7	854	1	848	CDS
hsa-miR-145	7	361	2	355	CDS
hsa-miR-1469	7	138	1	132	CDS
hsa-miR-1539	7	7	1	1	CDS
hsa-miR-1825	8	212	2	205	CDS
hsa-miR-1915	8	42	1	35	CDS
hsa-miR-198	8	372	2	365	CDS
hsa-miR-199a-5p	7	213	1	207	CDS
hsa-miR-199b-5p	7	213	1	207	CDS
hsa-miR-206	8	857	2	850	CDS
hsa-miR-22	8	104	2	97	CDS
hsa-miR-22*	7	588	2	582	CDS
hsa-miR-224	9	609	1	601	CDS
hsa-miR-299-3p	7	659	2	653	CDS
hsa-miR-302a	7	295	1	289	CDS
hsa-miR-302b	7	295	1	289	CDS
hsa-miR-302c	7	295	1	289	CDS
hsa-miR-302d	7	295	1	289	CDS
hsa-miR-302e	7	295	1	289	CDS
hsa-miR-335*	7	491	1	485	CDS
hsa-miR-338-3p	7	232	2	226	CDS
hsa-miR-33a*	8	496	1	489	CDS
hsa-miR-33b*	7	211	1	205	CDS
hsa-miR-342-5p	7	577	2	571	CDS
hsa-miR-345	7	392	1	386	CDS
hsa-miR-361-3p	8	43	2	36	CDS
hsa-miR-411	7	596	1	590	CDS
hsa-miR-424	7	165	1	159	CDS
hsa-miR-432	7	781	2	775	CDS
hsa-miR-452	8	433	1	426	CDS

hsa-miR-486-5p	11	566	2	556	CDS
hsa-miR-497	7	165	1	159	CDS
hsa-miR-505*	7	62	1	56	CDS
hsa-miR-519a	8	294	2	287	CDS
hsa-miR-519b-3p	8	294	2	287	CDS
hsa-miR-519c-3p	8	294	2	287	CDS
hsa-miR-520a-5p	7	371	2	365	CDS
hsa-miR-524-3p	7	330	1	324	CDS
hsa-miR-525-3p	7	330	1	324	CDS
hsa-miR-525-5p	7	371	2	365	CDS
hsa-miR-526b	8	884	1	877	CDS
hsa-miR-532-5p	9	149	1	141	CDS
hsa-miR-548g	7	434	2	428	CDS
hsa-miR-562	7	866	2	860	CDS
hsa-miR-604	8	127	2	120	CDS
hsa-miR-604	7	787	1	781	CDS
hsa-miR-613	7	857	2	851	CDS
hsa-miR-622	7	893	2	887	CDS
hsa-miR-622	7	452	2	446	CDS
hsa-miR-628-5p	7	393	2	387	CDS
hsa-miR-634	7	233	2	227	CDS
hsa-miR-637	7	388	1	382	CDS
hsa-miR-637	7	580	2	574	CDS
hsa-miR-644	7	751	1	745	CDS
hsa-miR-646	7	156	1	150	CDS
hsa-miR-646	7	776	2	770	CDS
hsa-miR-663	7	328	1	322	CDS
hsa-miR-665	7	716	2	710	CDS
hsa-miR-668	7	274	1	268	CDS
hsa-miR-668	7	606	2	600	CDS
hsa-miR-671-3p	7	312	2	306	CDS
hsa-miR-675	7	132	2	126	CDS
hsa-miR-758	7	625	2	619	CDS
hsa-miR-767-5p	8	508	2	501	CDS
hsa-miR-877*	7	886	1	880	CDS
hsa-miR-877*	7	241	2	235	CDS
hsa-miR-886-3p	7	123	2	117	CDS
hsa-miR-888	7	724	1	718	CDS
hsa-miR-892b	7	271	1	265	CDS
hsa-miR-922	7	166	1	160	CDS
hsa-miR-93*	7	394	2	389	CDS
hsa-miR-940	7	423	1	417	CDS
hsa-miR-943	7	391	1	385	CDS
hsa-miR-1285	7	927	2	921	3UTR
hsa-miR-1286	7	1068	1	1062	3UTR
hsa-miR-1291	8	981	1	974	3UTR
hsa-miR-129-5p	7	1072	2	1066	3UTR
hsa-miR-1324	9	948	1	940	3UTR
hsa-miR-141*	10	954	1	945	3UTR
hsa-miR-185*	7	969	2	963	3UTR
hsa-miR-188-5p	7	1017	1	1011	3UTR
hsa-miR-20b*	7	1001	2	995	3UTR
hsa-miR-328	8	982	1	975	3UTR
hsa-miR-33b*	8	933	1	926	3UTR

hsa-miR-340*	7	1034	1	1028	3UTR
hsa-miR-450b-5p	7	1071	1	1065	3UTR
hsa-miR-485-5p	11	987	1	977	3UTR
hsa-miR-485-5p	7	921	1	915	3UTR
hsa-miR-491-5p	7	1172	2	1162	3UTR
hsa-miR-507	7	1071	1	1065	3UTR
hsa-miR-512-5p	7	897	1	891	3UTR
hsa-miR-515-3p	7	932	2	926	3UTR
hsa-miR-517*	7	991	2	985	3UTR
hsa-miR-518d-5p	9	991	1	983	3UTR
hsa-miR-518e*	9	991	1	983	3UTR
hsa-miR-518f*	9	991	1	983	3UTR
hsa-miR-519a*	9	991	1	983	3UTR
hsa-miR-519b-5p	9	991	1	983	3UTR
hsa-miR-519c-5p	9	991	1	983	3UTR
hsa-miR-519e	7	932	2	926	3UTR
hsa-miR-520c-5p	9	991	1	983	3UTR
hsa-miR-522*	9	991	1	983	3UTR
hsa-miR-523*	9	991	1	983	3UTR
hsa-miR-526a	9	991	1	983	3UTR
hsa-miR-550	8	932	1	925	3UTR
hsa-miR-612	8	927	2	920	3UTR
hsa-miR-625*	7	1052	1	1046	3UTR
hsa-miR-661	8	930	1	923	3UTR
hsa-miR-767-3p	7	995	1	989	3UTR
hsa-miR-873	9	1067	1	1059	3UTR
hsa-miR-920	8	911	2	904	3UTR

Table S2 The growth of subcutaneous tumors in NOD/SCID mice

**Stably transfected cells**	**Mice examined**	**Mice with tumor**	**Tumor sizes (mm^3, mean ± SD)**	**Tumor weights (mg, mean ± SD)**
H7-miR-224	6	6 (100%)	1848.0 ± 268.7	2746.8 ± 657.2
H7-GFP	6	6 (100%)	1388.9 ± 294.1	1783.8 ± 503.6
H7-GNMT/miR-224	6	6 (100%)	1006.2 ± 276.2	1077.6 ± 317.1
H7-GNMT	6	6 (100%)	847.7 ± 168.4	870.2 ± 234.0

Table S3. Main clinical and histopathologic features of 78 patients with hepatocellular carcinoma.

Clinicopathological feature	Variable	No. of cases (%)
Mean age ± SD, years (range)	60.5 ± 14.6 (13-85)	78 (100)
Gender	male	39 (50)
	female	39 (50)
Viral infection	HBV	40 (51)
	HCV	20 (26)
	NBNC	18 (23)
Cirrhosis	No	42 (54)
	Yes	36 (46)
AFP	≤100 ng/ml	40 (51)
	>100 ng/ml	38 (49)
Tumor size	≤3 cm	15 (19)
	>3cm	63 (81)
Tumor grade*	I	3 (4)
	II	18 (23)
	III–IV	57 (73)
TNM stage	I	37 (47)
(AJCC and UICC, 7th ed.)	II	17 (22)
	IIIA	10 (13)
	IIIB	10 (13)
	IIIC	2 (2.5)
	IV	2 (2.5)

Abbreviations: HBV, patients with hepatitis B virus surface antigen (HBs Ag); HCV, patients with antibody to HCV; NBNC, patients without HBs Ag and antibody to HCV; AFP, serum α-fetoprotein level.

* The tumor grade was divided into three groups: well (grade I), moderately (grade II), and poorly differentiated HCC (grades III and IV).

Table S4. Primer sequences and Taqman assays

Name	Items	Sequence/ Assay ID
pCMV5a-F	Clone-Primer	5`-AGCTCGTTTAGTGAACCGTCAG-3`
pCMV5a-R	Clone-Primer	5`-TCGACTGGTACCGATATCAGATC-3`
GNMTmt-F	Clone-Primer	5`- CTACTATAAGTCAGATCTCACCAAGGACGTC -3`
GNMTmt-R	Clone-Primer	5`- CGTCCTTGGTGAGATCTGACTTATAGTAGAT -3`
Xho1-CDS-F	Clone-Primer	5`-CCGCTCGAGGCGGCGCGCG-3`
Not1-CDS-R	Clone-Primer	5`-ATAAGAATGCGGCCGCCCAGGACGCTGTGC-3`
GNMT-F	qPCR-Primer	5`-ACTGGATGACTCTGGACAA-3`
GNMT-R	qPCR-Primer	5`-ACTGAGGATGTGGTCGT-3`
TBP-F[a]	qPCR-Primer	5`-TGCACAGGAGCCAAGAGTG-3`
TBP-R[a]	qPCR-Primer	5`-CACATCACAGCTCCCCACC-3`
API-5-F[b]	qPCR-Primer	5`-TAGTGGGTTTGGAGAAGTTC-3`
API-5-R[b]	qPCR-Primer	5`-TCACTTGATAGGCATCTTTATG-3`
SMAD4-F[c]	qPCR-Primer	5`-AGGATCAGTAGGTGGAATAG-3`
SMAD4-R[c]	qPCR-Primer	5`-TCTAAAGGTTGTGGGTCTGC-3`
hsa-miR-224	TaqMan Assay	477986_mir
RNU48	TaqMan Assay	001006
mmu-miR-224	TaqMan Assay	mmu482610_mir
snoRNA202	TaqMan Assay	001232
hGNMT-F	qPCR-Primer	5`-GCAGCCTTCGGAGGTAAGTG-3`
hGNMT-R	qPCR-Primer	5`-CTAGCAGTCCCTGGGCAGAG-3`
mGNMT-F	qPCR-Primer	5`-GCCTACGTTCCCTGCTACTT-3`

mGNMT-R	qPCR-Primer	5`-GCATTTGGGTGCAGATGTGG-3`
Collagen I-F	qPCR-Primer	5`-AAGAGGCGAGAGAGGTT-3`
Collagen I-R	qPCR-Primer	5`-CCTTTGGGACCAGCATC-3`
α-SMA-F	qPCR-Primer	5`-ATTCAGGCTGTGCTGTC-3`
α-SMA-R	qPCR-Primer	5`-TCTCACGCTCGGCAGTA-3`
TGF-β	qPCR-Primer	5`-CCAAAGACATCTCACACAGTA-3`
TGF-β	qPCR-Primer	5`-GCCACTCAGGCGTATCA-3`
β2m-F	qPCR-Primer	5`-ATCCTGGCTCACACTGAATTCA-3`
β2m -R	qPCR-Primer	5`-TGCTTAACTCTGCAGGCGTATG-3`

a. Gur-Dedeoglu B, Konu O, Bozkurt B, Ergul G, Seckin S, Yulug IG. Identification of endogenous reference genes for qRT-PCR analysis in normal matched breast tumor tissues. Oncology research 2009;17:353-65.

b. Wang Y, Lee AT, Ma JZ, Wang J, Ren J, Yang Y, Tantoso E, Li KB, Ooi LL, Tan P, Lee CG. Profiling microRNA expression in hepatocellular carcinoma reveals microRNA-224 up-regulation and apoptosis inhibitor-5 as a microRNA-224-specific target. The Journal of biological chemistry 2008;283:13205-15.

c. Wang Y, Ren J, Gao Y, Ma JZ, Toh HC, Chow P, Chung AY, Ooi LL, Lee CG. MicroRNA-224 targets SMAD family member 4 to promote cell proliferation and negatively influence patient survival. PloS one 2013;8:e68744.

Figure S1 The putative miR-224, miR-93* and miR-491-5p-response element in human GNMT

67 hsa-miRNA	
hsa-miR-100	hsa-miR-213
hsa-miR-106b	hsa-miR-216
hsa-miR-107	hsa-miR-216a
hsa-miR-10a	hsa-miR-221
hsa-miR-10b	hsa-miR-222
hsa-miR-125b	hsa-miR-224
hsa-miR-127-3p	hsa-miR-23a
hsa-miR-128b	hsa-miR-294
hsa-miR-130b	hsa-miR-301a
hsa-miR-135a	hsa-miR-30d
hsa-miR-137	hsa-miR-324
hsa-miR-143	hsa-miR-33
hsa-miR-151	hsa-miR-362
hsa-miR-155	hsa-miR-373
hsa-miR-15a	hsa-miR-374
hsa-miR-15b	hsa-miR-375
hsa-miR-16	hsa-miR-376a
hsa-miR-17	hsa-miR-382
hsa-miR-18	hsa-miR-490-3p
hsa-miR-182	hsa-miR-491
hsa-miR-182*	hsa-miR-500
hsa-miR-183	hsa-miR-519
hsa-miR-185	hsa-miR-519d
hsa-miR-186	hsa-miR-527
hsa-miR-18a	hsa-miR-550a
hsa-miR-18b	hsa-miR-590-5p
hsa-miR-195	hsa-miR-602
hsa-miR-19a	hsa-miR-615-5p
hsa-miR-19b	hsa-miR-657
hsa-miR-205	hsa-miR-9
hsa-miR-207	hsa-miR-92
hsa-miR-20a	hsa-miR-93
hsa-miR-21	hsa-miR-96
hsa-miR-210	

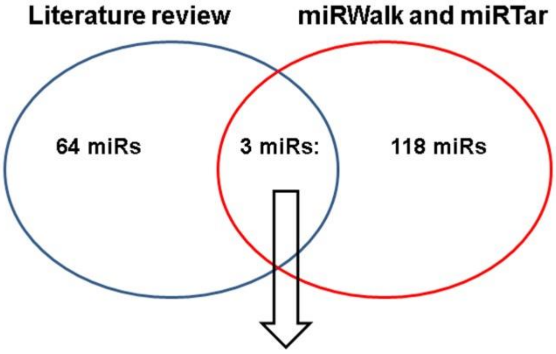

The miRNAs were predicted to target CDS and 3'UTR of GNMT gene

MicroRNA	Seed length	Start to end[#]	Sequence	Region
hsa-miR-224	9	601-609	CAAGUCACU	CDS
[&]hsa-miR-93*	6	389-394	CUGCUG	CDS
hsa-miR-491-5p	6	1167-1172	GUGGGG	3'UTR

GNMT transcription start site as 1

& Borel, Konstantinova et al. (2012) has been reported up-regulation of miR-93 in HCC.

Figure S2

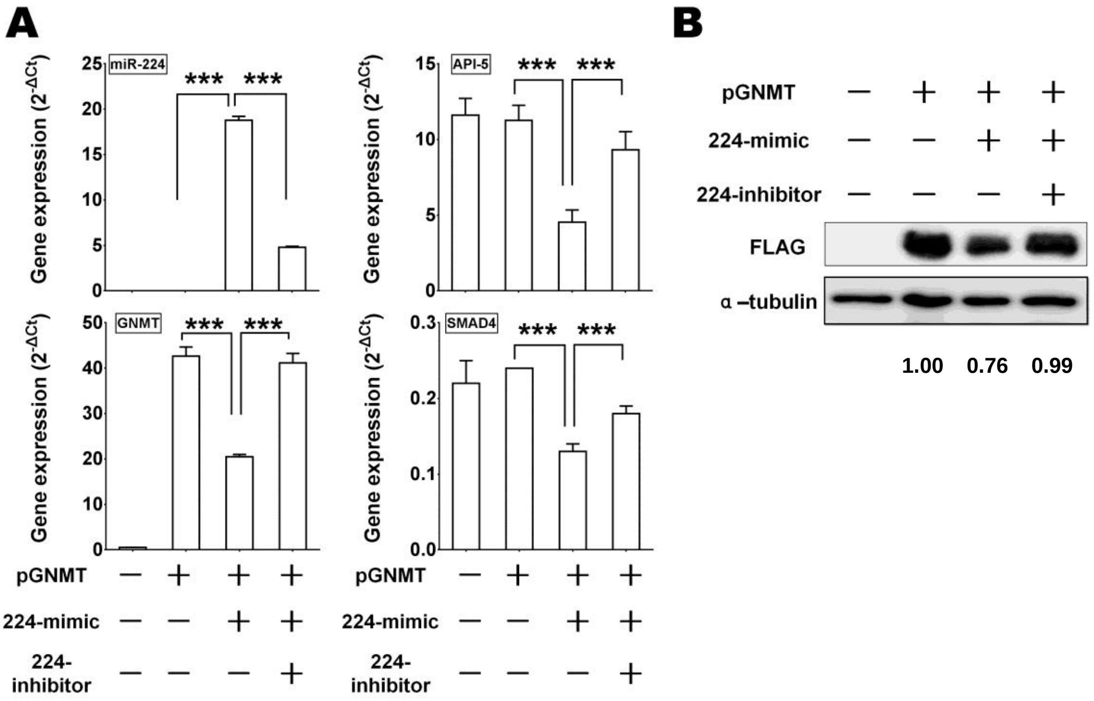

Figure S2. The exogenous GNMT was suppressed by miR-224. (A) Relative miR-224 and GNMT transcript measured with qPCR in HEK293T cells transiently transfected with a control group (eGFP plasmid, 50 nM NC and NC inhibitor) or pGNMT plasmid, 224-mimic, 224-mimic/224-inhibitor. These averages are for the three replicates shown on each array. The gene expression level of each sample was normalized to the level of TBP (TATA-binding protein) RNA. API-5 and SMAD4 served as positive controls to ensure the validity of 224-mimic and 224-inhibitor activity. (B) GNMT protein expression was measured by western blotting in HEK293T cells transfected with NC or pGNMT plasmid, 224-mimic and 224-mimic/224-inhibitor. α-tubulin was used as internal control.

Figure S3

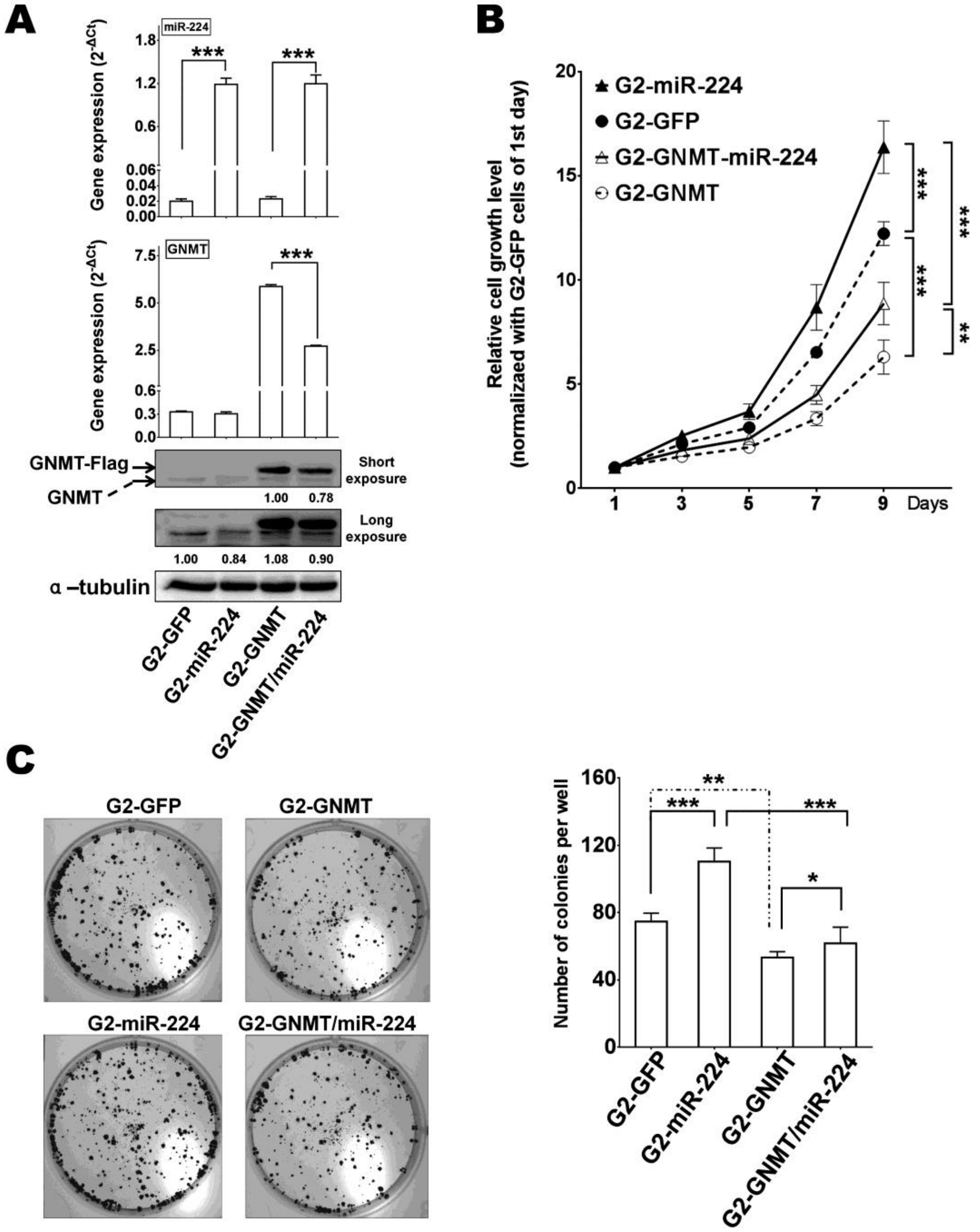

Figure S3. Effect of miR-224/GNMT on HepG2 cell line growth in vitro. (A) The GNMT and miR-224 transcript levels in HepG2 infected with lentivirus expressing miR-224 and GNMT were measured by qPCR (upper panel). ****P*

< 0.001 compared with cells infected with lentivirus expressing eGFP and scramble miR-224. GNMT protein expressions examine by western blotting (bottom panel). (B) The proliferation activity of HepG2 as measured by the alamarBlue assay at the time indicated. The absorbance of cells at day 1 was used as quantitative baseline. The expression level of each sample was normalized to the average of that of the control group. **$P < 0.01$, ***$P < 0.001$. (C) The colony formation was quantified by staining crystal violet. 1500 cells were seeded into each well of 6 well plates and incubated for 21 days.

Figure S4

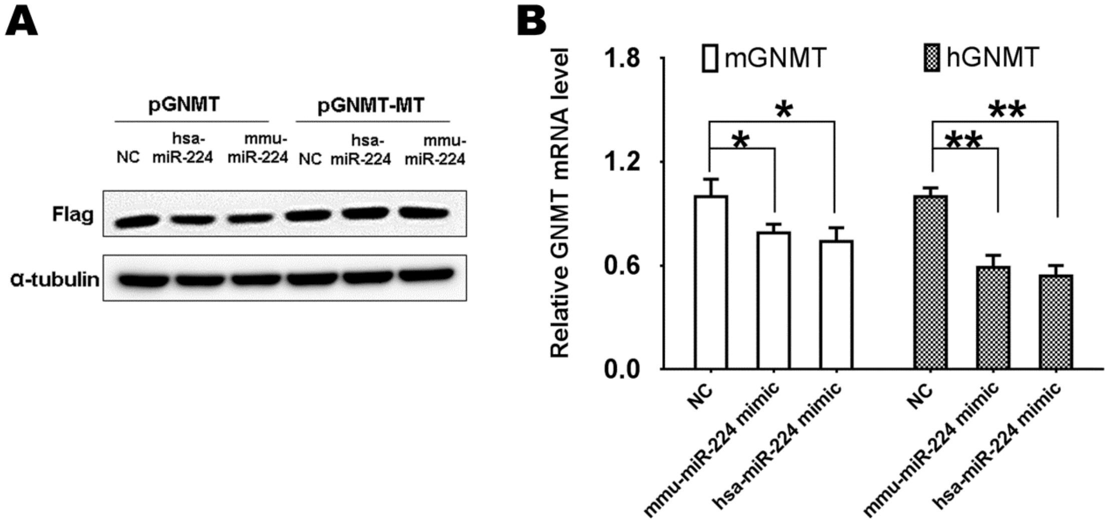

Figure S4. GNMT was regulated by miR-224 in human and mouse cell lines. (A) GNMT protein expression was measured by western blotting in HEK293T cells transiently cotransfected with pGNMT/mmu-miR-224 mimic, pGNMT/hsa-miR-224 mimic, pGNMT-MT/mmu-miR-224 mimic and pGNMT-MT/hsa-miR-224 mimic. α-tubulin was used as internal control. (B) Relative mouse and human GNMT transcript measured with qPCR in Hepa 1-6 cells transiently cotransfected with pGNMT/control group (50 nM NC), pGNMT/mmu-miR- 224 mimic and pGNMT/hsa-miR-224 mimic. Data were normalized to expression levels of the mouse β2m and huma TBP. The relative expression fold of GNMT mRNA normalized to control group, respectively.

Figure S5

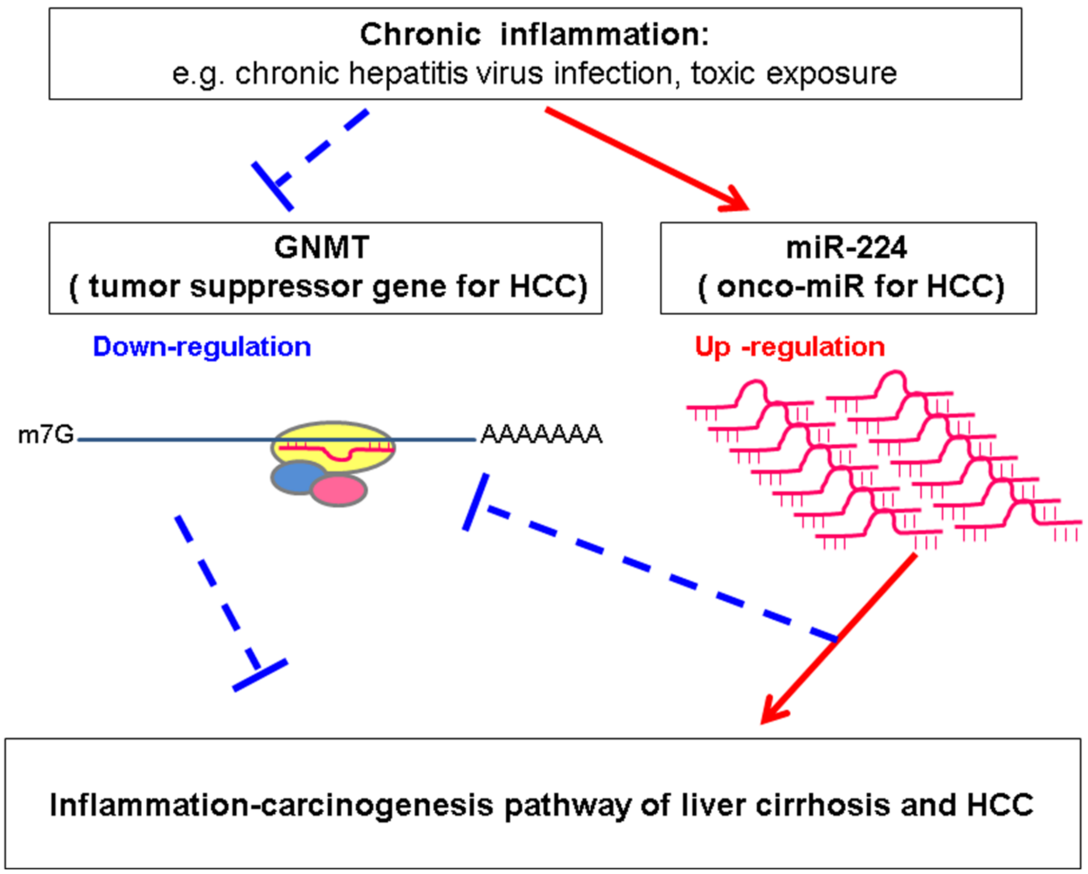

Figure S5

A scheme of the mechanism of GNMT down-regulation of chronic hepatitis virus infection and toxic exposure induced-inflammation response and miR-224 over-expression in liver cirrhosis and HCC.

Figure S6

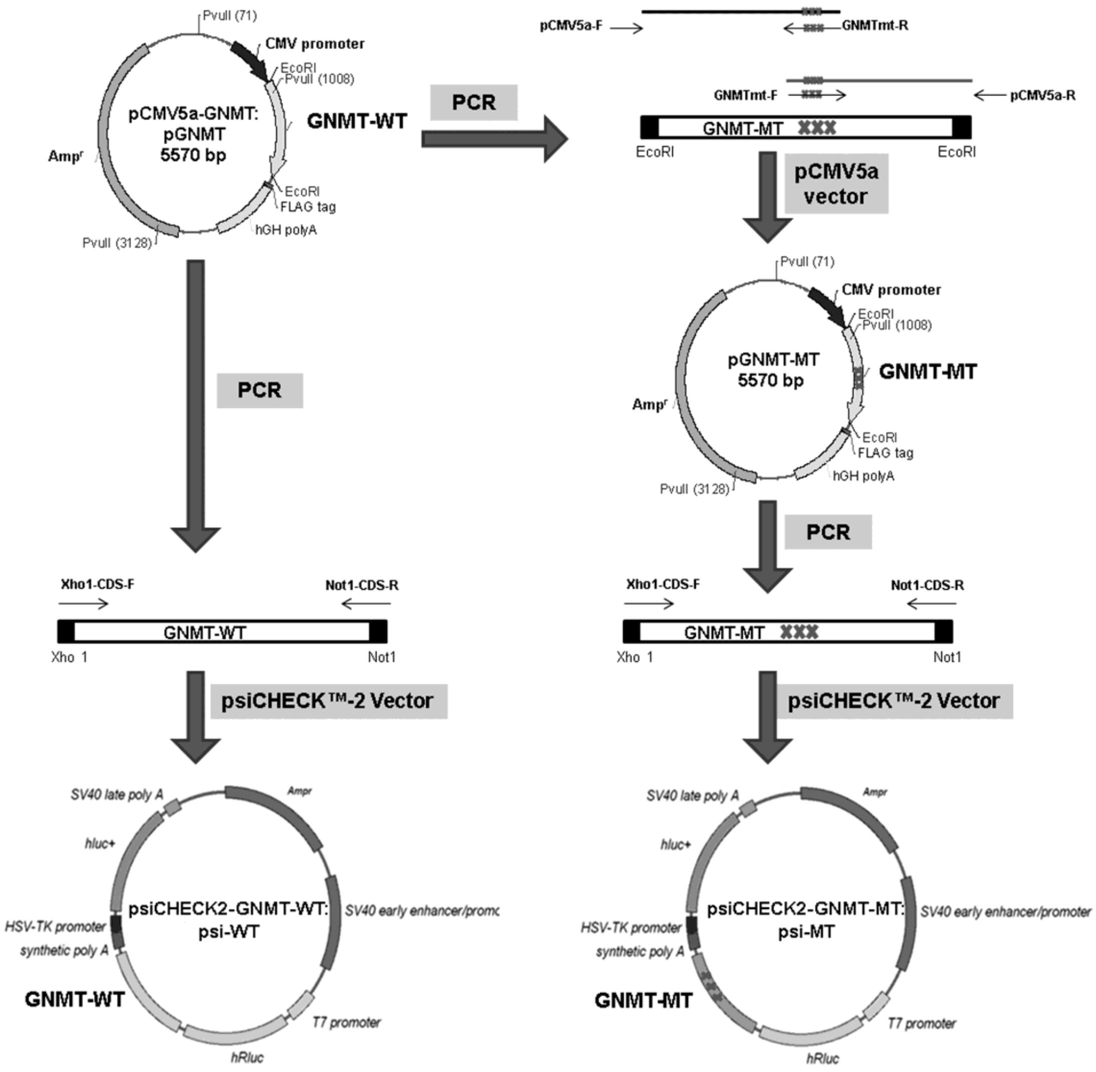

Figure S6. Construction of GNMT-MT, psiCHECK2-GNMT-WT (psi-WT) and psiCHECK2-GNMT-MT (psi-MT) plasmids. Molecular constructs were made in plasmid-pFLAG-CMV-5 and pSI-CHECK-2 by cloning a single restriction enzyme site (EcoRI) and the silent mutant (MT) GNMT cDNA (GNMT-MT, 0.9 kb) sequence and cloning behind the *Renilla* luciferase in the *Xho*I and *Not*I restrictions sites, GNMT-WT and GNMT-MT sequences from pGNMT and pGNMT-MT plasimds and named as psiCHECK2-GNMT-WT (psi-WT) and psiCHECK2-GNMT-MT (psi-MT).

Original picture for western blot

Pictures of main body	Original pictures

Fig. 2D

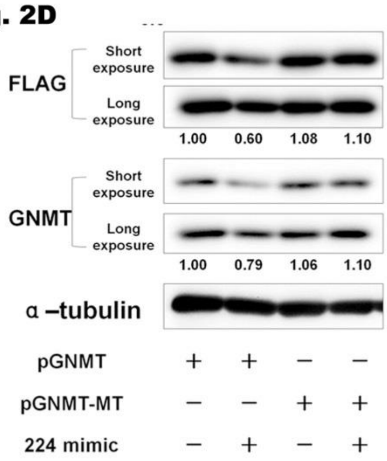

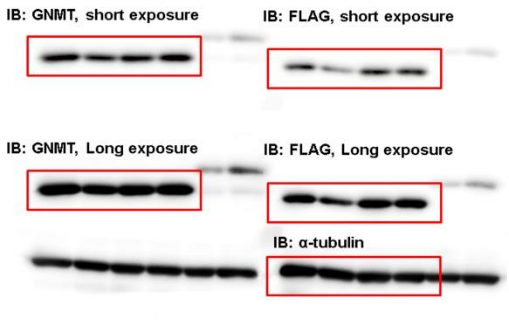

Fig. 3B

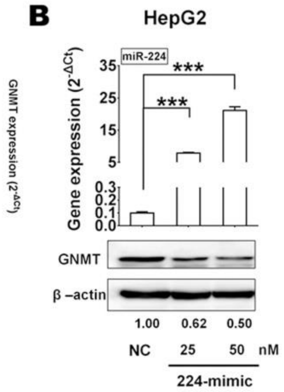

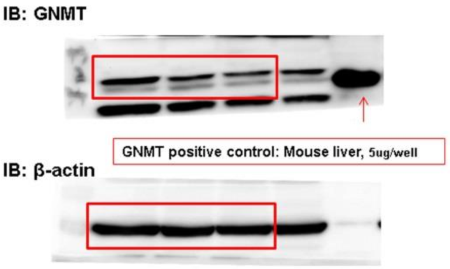

Cell line	HepG2	HepG2	HepG2	HepG2
Treatment	NCm	224 m	224 m	224 m
mim conc.	0	25	50	100
NC conc.	50	25	0	0
Fold change	1.00	0.74	0.65	0.73

Fig. 3C

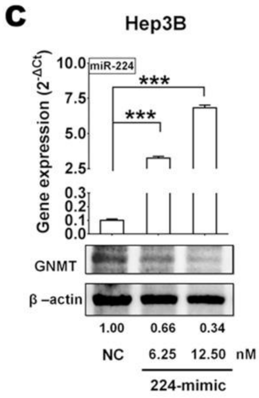

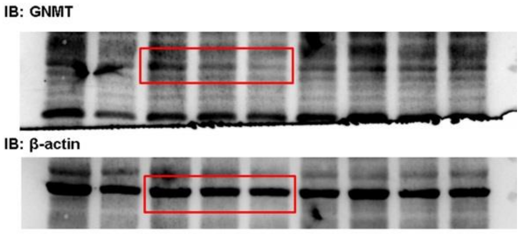

Cell line	Hep3B	Hep3B	Hep3B	Hep3B	Hep3B	Hep3B	Hep3B	Hep3B	Hep3B
Treatment	TKO	NT	NC	224 m	224 m	224 m	224 m	224 m	224 m
mim conc.			0	6.25	12.5	25	50	100	200
NC conc.			50	43.75	37.5	25	0		
Fold change			1.00	0.72	0.48	0.70	0.72	0.77	0.81

Pictures of main body | Original pictures

Fig. 3D

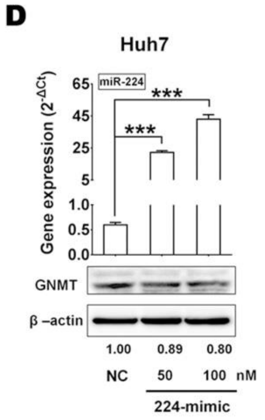

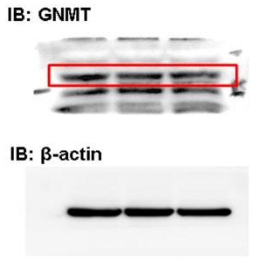

Fig. 3E

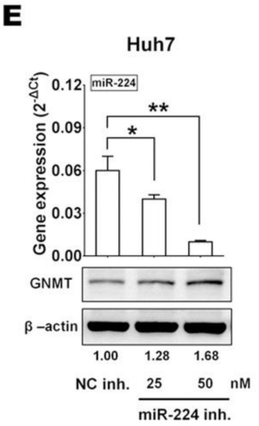

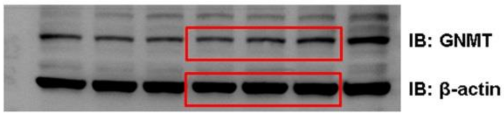

Cell line	Huh7	Huh7	Huh7	Huh7	Huh7	Huh7	Huh7
Treatment	NT	Mock	NCi	NCi.	224 inh.	224 inh.	224 inh.
Inhibitor conc. (nM)	0	0	0	0	25	50	200
NC conc. (nM)	0	0	200	50	25	0	0
Fold change	1.13	0.98	0.97	1.00	1.32	1.72	1.95

Fig. 4A and Fig. S3A

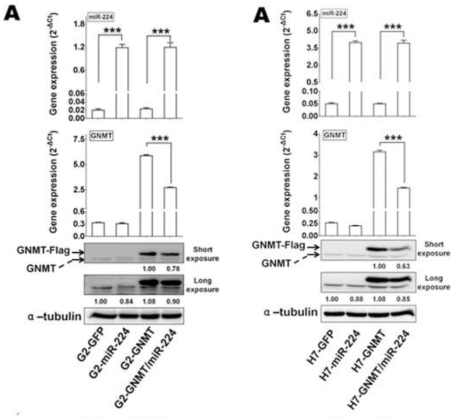

Fig. S3A   Fig. 4A

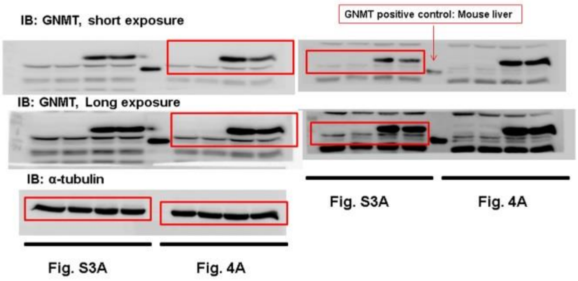

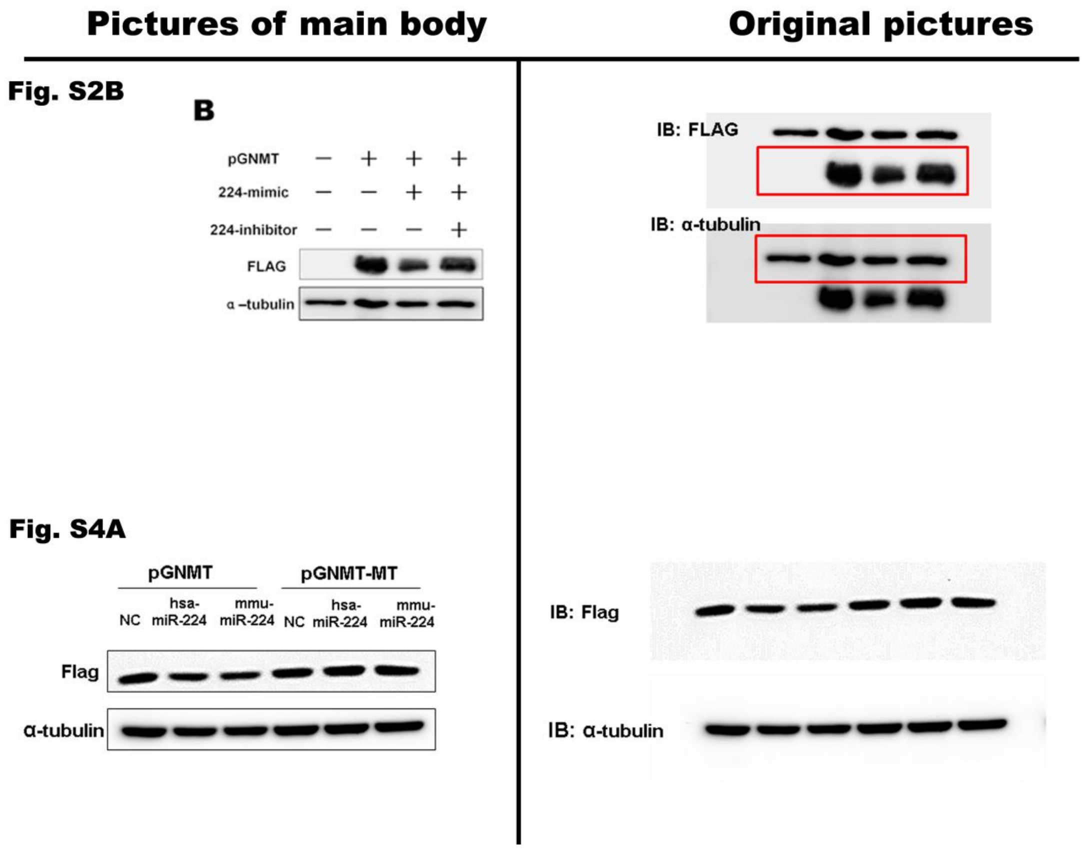

Original picture for western blot. Western blot analysis of figures in text corresponded with original data.
